# Transcriptional and proteomic analysis of the innate immune response to microbial stimuli in a model invertebrate chordate

**DOI:** 10.3389/fimmu.2023.1217077

**Published:** 2023-08-02

**Authors:** Assunta Liberti, Carla Pollastro, Gabriella Pinto, Anna Illiano, Rita Marino, Angela Amoresano, Antonietta Spagnuolo, Paolo Sordino

**Affiliations:** ^1^ Biology and Evolution of Marine Organisms (BEOM), Stazione Zoologica Anton Dohrn, Naples, Italy; ^2^ Department of Chemical Sciences, University of Naples Federico II, Naples, Italy; ^3^ Istituto Nazionale Biostrutture e Biosistemi-Consorzio Interuniversitario, Rome, Italy; ^4^ Biology and Evolution of Marine Organisms (BEOM), Stazione Zoologica Anton Dohrn, Sicily Marine Centre, Messina, Italy

**Keywords:** inflammatory response, innate immunity, *Ciona robusta*, marine invertebrates, microbial stimuli, transcriptional analysis, proteomic analysis, protein-protein interactome

## Abstract

Inflammatory response triggered by innate immunity can act to protect against microorganisms that behave as pathogens, with the aim to restore the homeostatic state between host and beneficial microbes. As a filter-feeder organism, the ascidian *Ciona robusta* is continuously exposed to external microbes that may be harmful under some conditions. In this work, we used transcriptional and proteomic approaches to investigate the inflammatory response induced by stimuli of bacterial (lipopolysaccharide -LPS- and diacylated lipopeptide - Pam2CSK4) and fungal (zymosan) origin, in *Ciona* juveniles at stage 4 of metamorphosis. We focused on receptors, co-interactors, transcription factors and cytokines belonging to the TLR and Dectin-1 pathways and on immune factors identified by homology approach (*i.e*. immunoglobulin (Ig) or C-type lectin domain containing molecules). While LPS did not induce a significant response in juvenile ascidians, Pam2CSK4 and zymosan exposure triggered the activation of specific inflammatory mechanisms. In particular, Pam2CSK4-induced inflammation was characterized by modulation of TLR and Dectin-1 pathway molecules, including receptors, transcription factors, and cytokines, while immune response to zymosan primarily involved C-type lectin receptors, co-interactors, Ig-containing molecules, and cytokines. A targeted proteomic analysis enabled to confirm transcriptional data, also highlighting a temporal delay between transcriptional induction and protein level changes. Finally, a protein-protein interaction network of *Ciona* immune molecules was rendered to provide a wide visualization and analysis platform of innate immunity. The *in vivo* inflammatory model described here reveals interconnections of innate immune pathways in specific responses to selected microbial stimuli. It also represents the starting point for studying ontogeny and regulation of inflammatory disorders in different physiological conditions.

## 1 Introduction

The ascidian *Ciona robusta* is a filter-feeding marine species belonging to Tunicata, the sub-phylum most closely related to Vertebrata within the phylum Chordata (1). This species commonly thrives in polluted habitats highly enriched in pathogenic and nonpathogenic bacteria, fungi and viruses, sensing either potentially infectious ones or establishing homeostatic relationships, respectively. *C. robusta* is a consolidated experimental system in a wide array of biological fields (2–5) including immunology since, like all invertebrates, it possesses only innate immunity as a defense strategy against infections (6). The absence of the adaptive immune system facilitates studies of the innate immune crosstalk with the environment and of the establishment and maintenance of homeostasis with nonpathogenic and “foreign” microorganisms. In recent years, this marine species has been exploited for studies of host-microbiome interactions within the digestive tract in relation to microbial settlement and mucus colonization, leading to the characterization of different elements of the gut environment, such as chitin-rich mucus and a subset of secreted immune effectors (7, 8). *Ciona* has also been used in the context of identifying and defining immunological memory as a consequence of sophisticated responses mediated by trained immunity after inflammatory challenges (9).

The study of inflammatory mechanisms is essential to understanding the role of the immune system in the interaction with microbiota (10), and as a sentinel in cellular and tissue homeostasis (11–13). When these conditions deviate from homeostasis, activation of inflammatory pathways can help to restore the physiological equilibrium (11). In *Ciona*, several genomic and transcriptional studies have focused on the role of the innate immune system in the inflammatory response after challenges mainly with lipopolysaccharide (LPS) (reviewed in (14)), a component of Gram-negative bacterial membrane that belongs to the category of pathogen-associated molecular patterns (PAMPs). The knowledge of the immune repertoire of *C. robusta* is based on domain sequence similarities and phylogenetic relationships with their vertebrate counterparts (14), while the immunological role was functionally confirmed at the protein level in only few cases (15–17).

Several classes of immune factors have been recognized in *C. robusta*, such as *i*) pathogen recognition receptors (PRRs) like the Toll-like receptors (TLRs) TLR1 and TLR2 (17, 18) and C-type lectin receptors (CLRs) like CD94 (19); *ii*) cytosolic lectins like galectins, collectins (i.e. mannose-binding lectins) and intelectins (14, 20–24); *iii*) molecules of the complement system like C3 and its receptor C3aR (15, 16, 25, 26); *iv*) cytokines like the three interleukin-17 family members IL17-1, IL17-2 and IL17-3, the interleukin receptor IL17-R (27), the tumor necrosis factor α (TNFα) (28), the transforming growth factor beta (TGF-β) (29) and the macrophage migration inhibitory factor (MIF) (30), *v*) defensins like the molecule against microbes A precursor (mamA) (31); and *vi*) cytokine induction mediators like the gene coding for the transcription factor nuclear factor kappa B (NF-κB) (17, 32). All these immune molecules are transcriptionally, and sometimes translationally, upregulated in hemocytes and pharynx following subtunical injection of LPS in the pharyngeal wall, the first portion of the digestive tract of adult individuals of *C. robusta* (14). Moreover, C3 and C3aR have been proven to exert a chemotactic activity on ascidian hemocytes (15, 16). Here, our analyses of mRNA and protein expression patterns included most of these molecules, as well as additional genes putatively involved in the inflammatory response that were identified by homology search analysis in the current study.

An important experimental advantage of adopting *C. robusta* in comparative immunology is the capacity to perform *in vitro* fertilization and rear thousands of transparent filter-feeding juveniles. Metamorphic stage 4 of juvenile development (1^st^ ascidian stage) is the first filter-feeding stage in the life cycle of *Ciona* (33), and can be used for investigating the inflammatory response to environmental microbial components at the early phase of immune system development and maturation. While the inflammatory response in adult ascidians usually involves stimulus injection in the body wall, stage 4 C*. robusta* juveniles can be exposed to resuspended, exogenous, and sometimes harmful compounds that can be ingested by filtration, thus reproducing a more natural physiological condition. In this work, this juvenile stage was used to explore the activation of key components of the innate immune response in the whole animal after exposure to different PAMPs.

The inflammatory molecules used in this study include components of bacterial [i.e., LPS and diacylated lipopeptide (Pam2CSK4)] and fungal (*i.e.*, zymosan, an insoluble preparation of *Saccharomyces cerevisiae* cell wall, consisting of β-glucans, mannans, mannoproteins and chitin) cell walls. Of note, the effect of Pam2CSK4 [present in both Gram-positive and Gram-negative bacteria (34, 35)] on *Ciona* immune response has not been previously investigated. In mammals, these stimuli are described to be agonists of TLRs and Dectin-1. Specifically, LPS is an agonist of human TLR4 (36, 37), Pam2CSK4 is an activator of TLR2/TLR6 heterodimer (38), and zymosan is an agonist of human TLR2 and Dectin-1 (39). In a previous study conducted by Sasaki and coworkers using *Ciona* adults, activation of TLRs was induced by zymosan and not by another lipopeptide, Pam3CSK4 (17).

Here, we gathered evidence in support of specific transcriptional and translational changes induced by PAMPs, and of possible interactions between molecules and pathways involved in the immune response, specifically TLRs and Dectin-1 pathways. In summary, this study depicts *C. robusta* as a unique chordate organism for studying factors and mechanisms that modulate immune activation and homeostasis, thus supporting its use as a viable experimental system in translational research and biotechnological approaches.

## 2 Materials and methods

### 2.1 Ethics statement and animal sampling

The research described herein was performed by using *Ciona* specimens collected in the Mar Piccolo of Taranto (Taranto, Italy, ~40°29’29”N, 17°17’55”E) and in the Fusaro lagoon (Naples, Italy, ~40°49’10”N, 14°03’28”E), in locations that are not privately-owned nor protected in any way, according to the authorization of Marina Mercantile (Decree of the President of the Republic (DPR) 1639/68, Sep. 19, 1980, confirmed on Jan. 10, 2000). The study did not involve mammalian or vertebrate subjects, or endangered or protected species, and was carried out in strict accordance with European (Directive 2010/63/EU) and Italian (Legislative Decree n. 26/2014) legislation for the care and use of animals for scientific purposes. *C. robusta* is considered an introduced species and is not regulated or protected by environmental agencies in Italy. The animal collection services contracted in this study maintain current permits and licenses for collection and distribution of marine invertebrates to academic institutions. No special permission was required to collect ascidians, and animal handling was in accordance with the guidelines of our academic institutions. Animals were recovered and brought to the laboratory alive and maintained in clean water with aeration, temperature control and properly fed. In accordance with general animal protocols, the least number of specimens required per experiment were utilized. Animal waste products were disposed appropriately.

### 2.2 Juvenile treatment and sample preparation for gene expression and proteomic analyses

To obtain stage 4 *Ciona* juveniles, *in vitro* fertilization was performed using eggs and spermatozoa surgically collected from the gonoducts of different animals that had been exposed to constant light to elicit gamete maturation. *In vitro* egg fertilization follows the procedure described previously (40), with the exception that 0.22 μM filtered seawater (FSW) was used and no sterilization step was performed. After the time required for egg fertilization (about 10 minutes), fertilized eggs were diluted in FSW in 150 mm Petri dishes and raised at 18°C. Eighteen hours (hr) later, when animal have reached the non-feeding swimming tadpole larval stage, batches containing at least 90% normally developed larvae were selected for inflammatory treatments. Then, larvae were gently transferred to 6-well plates containing 4 ml FSW and 600-700 larvae per well. Animals were let to grow O/N at 18°C, and the day after the FSW was replaced with fresh one to eliminate post-metamorphic stages not attached to the dish, or unsettled larvae not properly developed. When juveniles have reached stage 4 (33), 3-4 days post fertilization, they were ready to be treated with different inflammatory stimuli, such as LPS (Sigma #L2880), Pam2CSK4 (InvivoGen #tlrl-pm2s-1) and zymosan (InvivoGen #tlrl-zyn). We referred to literature for the concentration of each inflammatory agent that best suits the activation of the immune system. LPS is usually injected in adults in the quantity of 100 μg dissolved in marine solution (27–29), or resuspended in a cell line-based heterologous system at the concentration of 2.5 and 5 μg/ml (17). Here, we used LPS at an intermediate concentration of 10 μg/ml. The triacylated form Pam3CSK4 was tested on cell lines in a heterologous system at the concentration of 0.5 and 2 μg/ml (17). Here, we used Pam2CSK4 at 1 and 10 μg/ml concentrations. The third inflammatory agent, zymosan, was reported in *Ciona* as a modulator of TLR activation in an *in vitro* heterologous system at the concentration of 100 μg/ml (17). Here, we used it at 10 and 100 ug/ml concentration. Each compound was resuspended at the appropriate concentration in 4 ml of 0.22 μm FSW, and animals were treated with these solutions for 30 minutes (min), 2 hr and 4 hr, while control animals were exposed to normal FSW. At the end of each time treatment, samples for gene expression analysis were collected in RNA*later* by replacing the FSW supplemented with the microbial stimulus with 1 ml RNA*later* solution in each well. Sample storage followed the manufacturer’s instruction; briefly, after O/N incubation in RNA*later* at room temperature (RT), RNA*later* solution was removed and samples (whole animals still attached to the bottom of 6-well plates) were frozen at -20°C for long term storage until RNA extraction. In turn, samples for proteomic analyses were processed by removing FSW supplemented with the microbial stimulus followed by fast freezing in dry ice and storage at -80°C until protein extraction.

### 2.3 Gene expression analysis

#### 2.3.1 The identification of immune molecules

To search for new immunocompetent molecules, we used orthologous gene search and homology domain approaches to explore the *C. robusta* genome annotation databases ANISEED (v.2019) (41), NCBI (42) and UCSC Genome Browser (assembly ID: ci3 (43)). A combination of tools (e.g., PSI-BLAST (44), BLAST v.2.13.0 (45) and Jalview v.2.11.2.0 (46)) and resources (e.g., UniProt (47), InterPro (48) and SMART (49)) was used to compare protein functional and structural domains and to analyze the percentage of sequence identity of molecules of interest between *Ciona robusta* and *Homo sapiens*. The following protein domains were used as queries: *i*) C-type lectin (CTL) or carbohydrate-recognition domain (CRD) (CLECT); *ii*) interferon regulatory factor (IRF) domain (also known as tryptophan pentad repeat); and *iii*) immunoglobulin (Ig) and immunoglobulin-like (Ig-like) domains.

#### 2.3.2 RNA extraction

Total RNA was extracted from whole-body *Ciona* juveniles using RNAqueous™-Micro Total RNA Isolation Kit (Invitrogen #AM193) following manufacturer’s instructions. Briefly, juvenile samples collected in 6-well plates as described in the previous section, were allowed to thaw slowly before proceeding to RNA extraction. After adding lysis buffer, samples were detached by scraping using flat blade cell lifter, collected, and transferred to 1.5 ml microtube, and mechanically broken using an ultra sonicator (Branson) for 15 seconds (sec) at 20% of maximum power. RNA extracted was eluted in 20 μl elution buffer, and subsequently DNA contamination was eliminated by performing DNase step as described in the manufacturer’s procedure. RNA quality and quantity were evaluated through agar gel electrophoresis and NanoDrop spectrophotometer (ThemoFisher) reading, respectively.

#### 2.3.3 Quantitative reverse transcription PCR analysis

Single-stranded cDNA was synthesized from 1 μg of total RNA employing QuantiTect Reverse Transcription kit (Qiagen #205311). Gene expression was analyzed by quantitative reverse transcription PCR (RT-qPCR) on cDNA from control juvenile samples and juvenile samples exposed to the different microbial stimuli. Primer sequences for the genes to be examined are listed in **Supplementary Tables 1A, B**. Actin gene was used as reference for internal standardization.

The amplification efficiency of each RT-qPCR primer set was assessed employing 10-fold serial dilution of juvenile cDNA or it was already tested in a previous work, employing 10-fold serial dilution of cDNA synthetized from *Ciona* digestive tract (50) (**Supplementary Tables 1A, B**). RT-qPCR was performed according to the manufacturer’s recommendation with the Fast SYBR Green MasterMix (Applied Biosystems #4385612), 0.28 μM of each primer and 5 ng of cDNA per reaction. A denaturation step at 95°C for 20 sec, 40 amplification cycles (95°C for 1 sec and 60°C for 20 sec) and a Melt Curve step (95°C for 15 sec, 60°C for 1 min and 95°C for 15 sec) were employed. Reactions for each sample were performed in triplicate on five or six biological replicates. In order to calculate mRNA expression level (mRNA RQ) relative to the control sample of each fertilization, data were analyzed with Vii™ 7 Real-Time PCR software (Life Technologies) and quantified with the comparative *C_t_* method (2^-ΔΔ^ *^Ct^*) based on Ct values. Expression level of the selected genes is indicated as fold change. Results are represented as violin plots, showing the full distribution of the data, reported as value of the fold change of each sample (biological replicate), and the median for each condition analyzed. Significance of the relative 2^-ΔΔCt^ of each group (biological replicates n = 5 or 6), compared to the controls, was determined using ‘paired parametric t-test’. One-way ANOVA analysis, according to normality test results, performed on each gene, was employed to determine statistically significant differences between the three time points for each microbial stimulus treatment. The statistical analyses were performed using GraphPad PRISM software, version 9.3.1.

### 2.4 Targeted proteomics analysis

#### 2.4.1 Protein extraction and samples preparation for mass spectrometry analysis

Total protein extraction from whole-body *C. robusta* juveniles followed the same procedure used for RNA extraction (*paragraph 2.3.2*) with the exception that the juveniles were lysed in 2% CHAPS buffer (100 μl per well, Sigma #C9426) and mechanically broken using an ultra sonicator (Branson) for 30 sec at 20% of maximum power. Sample lysates were then centrifuged at 13,000 rpm for 15 min at 4°C, supernatants were collected in 1.5 ml microtubes and stored at -80°C. An aliquot of 30 μl of each protein lysate was subjected to the *in-solution* digestion protocol by reducing with 100 mM dithiothreitol (dissolved in 50 mM ammonium bicarbonate) to a final concentration of 20 mM and incubated for 60 min at 60°C. After cooling the protein solution at RT, the protein cysteines were alkylated by adding iodoacetamide to a final concentration of 40 mM, followed by incubation in the dark for 45 min at RT. A solution of formic acid was added at a final concentration of 1% to block the alkylation reaction and proteins precipitated by a chloroform/methanol/water precipitation protocol (51). Supernatants were removed and the pellets dried. Digestion of protein mixtures was carried out in 10 mM ammonium bicarbonate by using trypsin at 50:1 protein:enzyme mass ratio. The samples were incubated at 37°C for 16 hr, and the trypsin digestion stopped by acidification of the peptide’s mixture.

The peptides were dried under vacuum and finally resuspended in 50 μl of 0.1% HCOOH for a further desalting step using manually equipped tips with three Empore disc C18 (Merck, #66883-U) before the liquid chromatography-mass spectrometry tandem (LC-MS/MS) analysis in Multiple Reaction Monitoring ion mode (MRM).

#### 2.4.2 LC-MS/MS analysis

The peptide mixture was analyzed by LC-MS/MS analysis using a Xevo TQ-S (Waters, Milford, MA, USA) equipped with an ionkey coupled to an Acquity UPLC system (Waters, Milford, MA, USA). For each run, the peptide mixture (2 µl) was injected and separated on a BEH C18 peptide separation device (130Å, 1.7 µm, 150 µm X 50 mm) at 45°C with a flow rate of 3 µl/min using an aqueous solution (LC-MS grade) containing 2% ACN as a mobile phase A and 98% ACN of an aqueous solution as a mobile phase B, both acidified with 0.2% HCOOH. The gradient for the MRM method started with 7% buffer B for 5 min, from 5 to 40 min reached 50% buffer B to 95% buffer B during the next 2 min. The column was finally re-equilibrated to initial conditions for 4 min. The parameters of the MS source were as follow: 3900 V as ion spray voltage, 150°C interface heater temperature, 150 l/h gas flow with 7 bar nebulizer pressure.

MRM mass spectrometric analyses were performed in positive ion mode for the run time with 5 points per peak and 3 min dwell times. The cone voltage was set to 35 V. A range of 300-1000 m/z was preferentially selected as precursor or product ions.

#### 2.4.3 Informatics tools

The latest version of Skyline software (22.2 - 64 bit version MacCossLab Software, University of Washington, USA) (52) was used for *in silico* selection of peptides with proteotypic sequences for each selected protein. For each peptide, m/z precursor ion, m/z product ion, and relative collision energy were provided by Skyline (**Supplementary Table 2**). Seven and thirty amino acid-long tryptic proteotypic peptides, preferably without missed cleavages and devoid of methionine and cysteine residues, were chosen for the development of MRM assays. Sequences with a proline (P) on the C-terminal side of arginine (R) or lysine (K) or showing the NXT or NXS glycosidic consensus motif were also excluded. The six transitions of proteotypic peptides were selected for method development based on the y-fragment ions.

#### 2.4.4 Statistical analysis

The areas of extracted ion chromatograms of all proteotypic peptides for each protein were averaged to get a value representative of a specific protein. Such values were uploaded on Perseus software (53) used for the statistical analysis. Expression values of each protein were log2 transformed to obtain a Perseus matrix. Finally, heatmap of cluster analysis and principal component analysis (PCA) were performed by using the obtained Perseus matrix.

### 2.5 *In silico* analysis of potential interactions

To increase knowledge about the landscape of molecular dynamics triggered by inflammation, a protein-protein interaction map was generated using the STRING database (54). Multiple sequence queries were followed by evaluation of protein sequence identity by means of Multiple Sequence Alignment (MSA) in Jalview (46) and BlastP (44, 45). The resulting interaction networks were modified and merged with the Cytoscape v.3.9.0 software (55) in order to build a complete protein-protein interactome in which to connect all molecules whose gene expression was analyzed in this study. Among the applications available in Cytoscape “App Manager” for functional analyses of immune-related pathways, we installed and used the STRING Enrichment application, that is based mainly on KEGG (56), SMART, COMPARTMENTS (57), Panther (58, 59), AmiGO 2 (60) and QuickGO (61), Pfam (62), InterPRO and Reactome (63) databases. This application was used to retrieve annotations about compartmentation, Gene Ontology (GO), pathway analysis and domain analysis of the selected nodes of the map. The main findings are reported in the protein-protein interaction network.

## 3 Results

### 3.1 Experimental setup: concentration and exposure time to microbial stimuli

**Figure 1:**
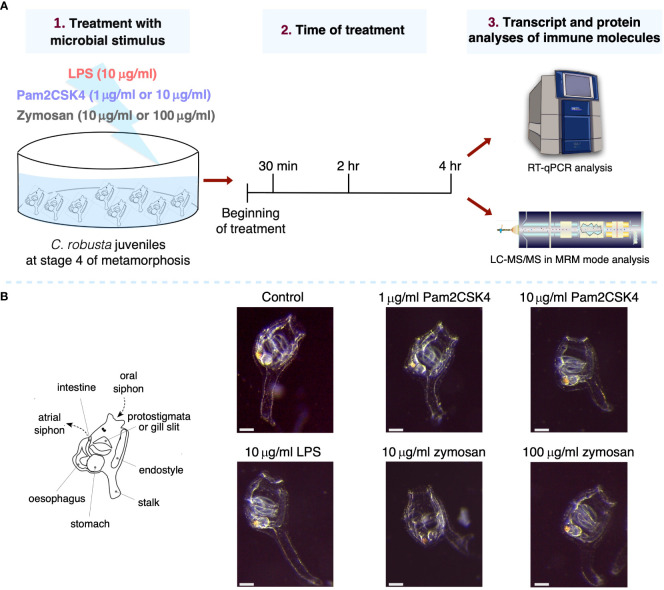
Experimental setup and effect on morphology of .*C*. *robusta* juveniles after 24 hours from the treatment. **(A)** Scheme of *C. robusta* juveniles’ treatment with different microbial stimuli, concentrations and duration, followed by quantification of immune molecules through gene expression (RT-qPCR technique) and protein level (LC-MRM/MC technique). **(B)** On the left, schematic representation of *C*. *robusta* juveniles at stage 4 showing the main anatomical features observed at this metamorphic stage. On the right, after 24 hours juveniles (exposed for 4 hours to diverse concentration of microbial stimuli) appear morphologically normal. Scale bar, 100 μm.

In this study, the effect of 30 min, 2 hr and 4 hr exposure to microbial stimuli in *C. robusta* juveniles has been investigated by evaluating changes in the expression levels of immune genes and proteins in the whole-body animal (**Figure 1A**). Also, juveniles were exposed for 4 hr at all selected concentrations (10 μg/ml LPS, 1 and 10 μg/ml Pam2CSK4, 10 and 100 μg/ml zymosan), raised in FSW for 24 hr, and then analyzed for survival and altered gross phenotypes. In all experimental conditions, juveniles were alive and morphologically normal, suggesting that the concentrations of the microbial stimuli did not trigger a strong immune response that could be lethal for the animals (**Figure 1B**).

### 3.2 Immune gene mining and expression

Previous studies have identified the main molecular actors of the innate immune system of *C. robusta*, revealing high-rate conservation of PRRs, cytokines and complement system molecules, to name a few. In our work, to widen the spectrum of molecules that could be responsive to the inflammatory stimuli here tested, more counterparts of genes involved in the inflammatory response of vertebrates were included. The genes encoding the tyrosine-protein kinase SYK and Nuclear factor of activated T-cells 5 (NFAT5), respectively co-interactor and transcription factor of PRR pathways, were identified by orthologous gene search and had already been annotated (http://www.aniseed.cnrs.fr). Then, more immune genes were uncovered by homology search analysis of *C. robusta* genome and proteome (**Table 1**). Specifically, we searched for molecules containing the CLECT and the Ig domains, since they are present in proteins with immune functions (64–67), and the IRF domain contained in the homonymous transcription factor involved in PRR pathways (68).

**Table 1:** *Ciona robusta* immune-related molecules identified by *in silico* analysis.

	Protein Name	NCBI Protein Acc. Number *C. robusta*	UniProtKB Acc. Number *C. robusta*	PSI-BLAST % identity	MSA % identity	Human Protein
**CLECT domain**	CLEC4M	XP_026689560.1	F7BDF2	34	23.40	CLEC4E
				33	21.40	CLEC4M
	CLEC4F	XP_002121230.2	H2XYQ6	23	18.40	CLEC4F
				23	20.73	CLEC4K
	MR	XP_026691626.1	F6TDB0	31	27.74	Macrophage mannose receptor 1 (MRC1)
				28	24.18	C-type mannose receptor 2 (MRC2)
**Ig-domain**	FAM187A (A/B)	XP_002120037.3	F6RE92	27	19.83	FAM187B
				25	19.60	FAM187A
	FN	AOO95899.1	F6TTH0 F6UFX8	24	21.30	Fibronectin
				21	20.77	Tenascin x
	FN-like	XP_002120276	F6TWX3	24	21.51	Fibronectin
				20	22.14	Tenascin x
	TYRO3	XP_002124888.2	F6U7K8	35	27.01	Tyro3
				33	26.02	Tyrosine-protein kinase receptor UFO
**IRF domain**	IRF4-like	NP_001071743.1	Q4H3B3	32	28.92	IRF4
				28	26.54	IRF5
	SYK	XP_002123000.1	F6QTW3	35	35.25	Syk
				33	33.33	Zap70
	NFAT5	XP_018666864.1	H2Y0S5	31	24.19	NFATC3
				29	26.07	Nfat5
	FAM136A	XP_002129880.1	A0A1W2WJY0	36	33.82	Fam136A
				24	17.99	NOP56

Protein names, accession numbers in NCBI and UniProtKB databases of C. robusta immune-related molecules are reported, together with the percentage of identity respect to human orthologs, investigated with both Protein Similarity Search (PSI-BLAST) database and Multiple Sequence Alignment Viewer application (MSA).

The CLECT domain allowed to identify the Dectin-1 CLR family 4 members M (CLEC4M) and F (CLEC4F), and Macrophage mannose receptor 1-like (MR). Ig- or Ig-like domains were found in FAM187A, fibronectin (FN), fibronectin like (FN-like), and tyrosine-protein kinase receptor 3 (TYRO3). TYRO3 is a receptor which is also characterized by FN and Tyrosine kinase catalytic domains and is involved in inflammation resolution (69, 70). FAM187A (Cirobu.g00001161) groups together with the ascidian *Botryllus schlosseri* Triggering receptor expressed on myeloid cells (TREM)-like 2 gene (TREML2) (Boschl.g00005277) in the “Gene Phylogeny” section of the ANISEED database. In human, the TREM2 pathway is expressed in various tissue macrophages, such as the microglia of the central nervous system, and increasing evidence suggests that TREM2 is involved in neuroinflammatory responses and neurodegenerative diseases like Alzheimer and Parkinson (71, 72) and contributes to mucosal inflammation in the digestive tract during the development of colitis in mice (73). The IRF domain was present in IRF-like gene. Furthermore, search for *C. robusta* NFAT5 led to the identification of FAM136A, a mitochondrial protein conserved across metazoans (74) whose human orthologue shows a correlation with Meniere’s disease, an inner ear problem with an autoimmune condition (75).

In detail, the effect of LPS, Pam2CSK4 and zymosan has been tested on the activity of the following *Ciona* factors: *i*) immune receptors such as TLR1, TLR2, CLEC4M, CLEC4F and MR; *ii*) Ig-like V-type domain-containing proteins as FAM187A, FN, FN-like and TYRO3; *iii*) the co-interactor SYK; *iv*) transcription factor coding genes NF-κB, IRF-like and NFAT5; *v*) FAM136A; *vi*) cytokines IL17-1, IL17-2, IL17-3, and their receptor IL17R, TNFα, TGFβ and MIF; *vii*) defensins like mamA and *viii*) complement system genes C3 and C3aR. The results obtained for each stimulus are described below. For sake of simplicity in the presentation, FAM136A and IL17R were included in the categories of transcription factors and cytokine signaling, respectively, besides their different roles.

#### 3.2.1 LPS: effects on TLR2 and TYRO3 genes

**Figure 2:**
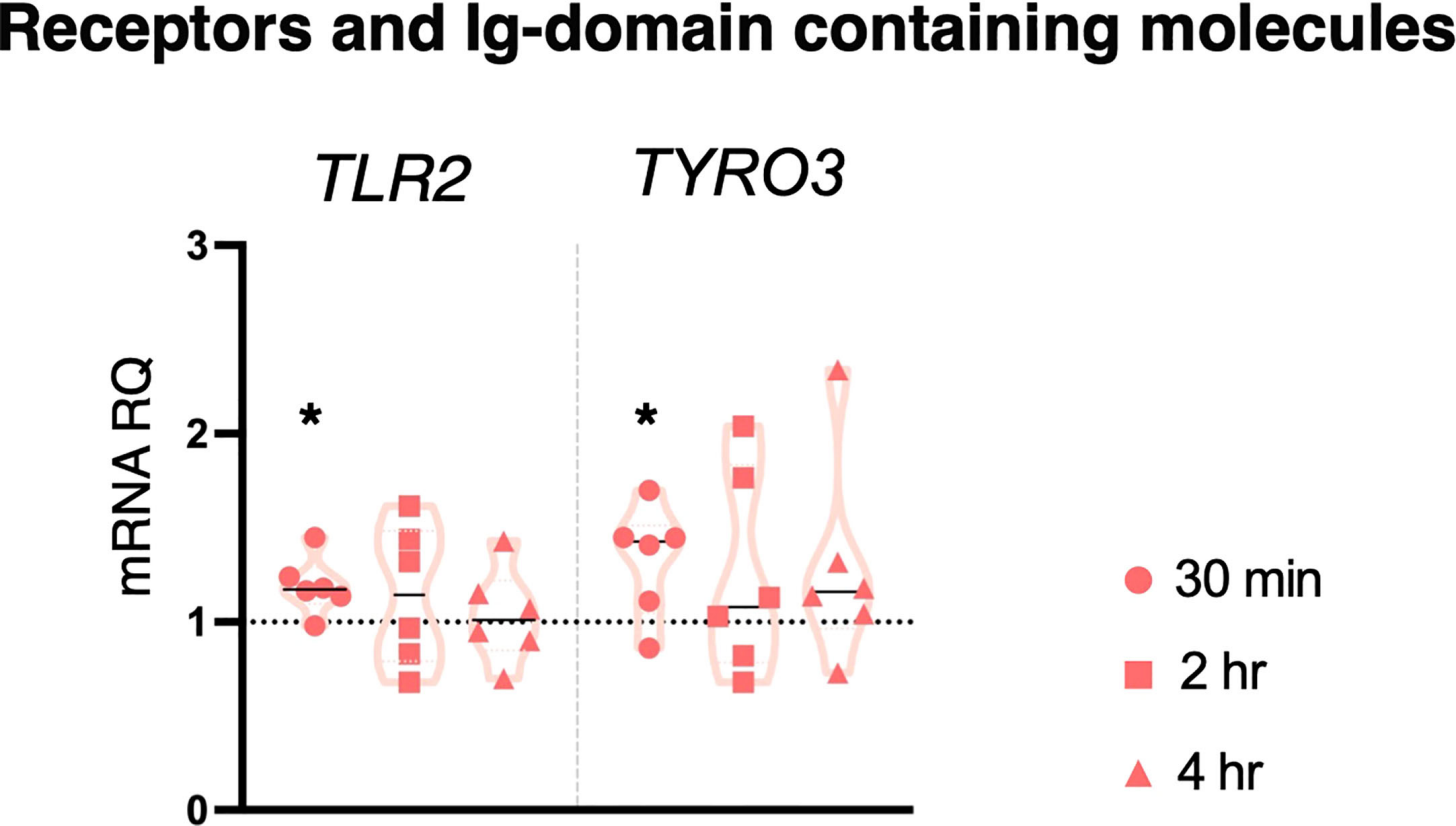
Effect of 10 μg/ml LPS treatment on gene expression. *C. robusta* juveniles at stage 4 of metamorphosis treated with 10 μg/ml LPS show significant changes in gene expression of *TLR2* and *TYRO3*, detected by RT-qPCR, after 30 min treatment. Truncated violin plots represent the distribution and the density of numerical data of gene expression reported as fold changes (2^-ΔΔCt^) of mRNA Relative Quantification (mRNA RQ) compared to the corresponding control samples (no treated juveniles), that are reported as dotted black line. The black lines in each violin plots indicate the median of data set (n = 6, biological replicates). Statistical methods: paired samples t-test, significance indicated by black asterisk. (*p. value < 0.05).

LPS is the inflammatory agent more widely used in *Ciona* adults (by injection). Here, 10 μg/ml LPS did not induce a significant immune response in stage 4 juveniles, as shown by lack of changes in the expression of most genes (**Supplementary Figure 1**) apart from the upregulation of *TLR2* and *TYRO3* after 30 min treatment (**Figure 2**). Thus, the modest activation of an immune response following LPS infection is rapidly resolved.

#### 3.2.2 Pam2CSK4: effects on genes encoding for PRRs, transcriptional factors, and cytokines

**Figure 3:**
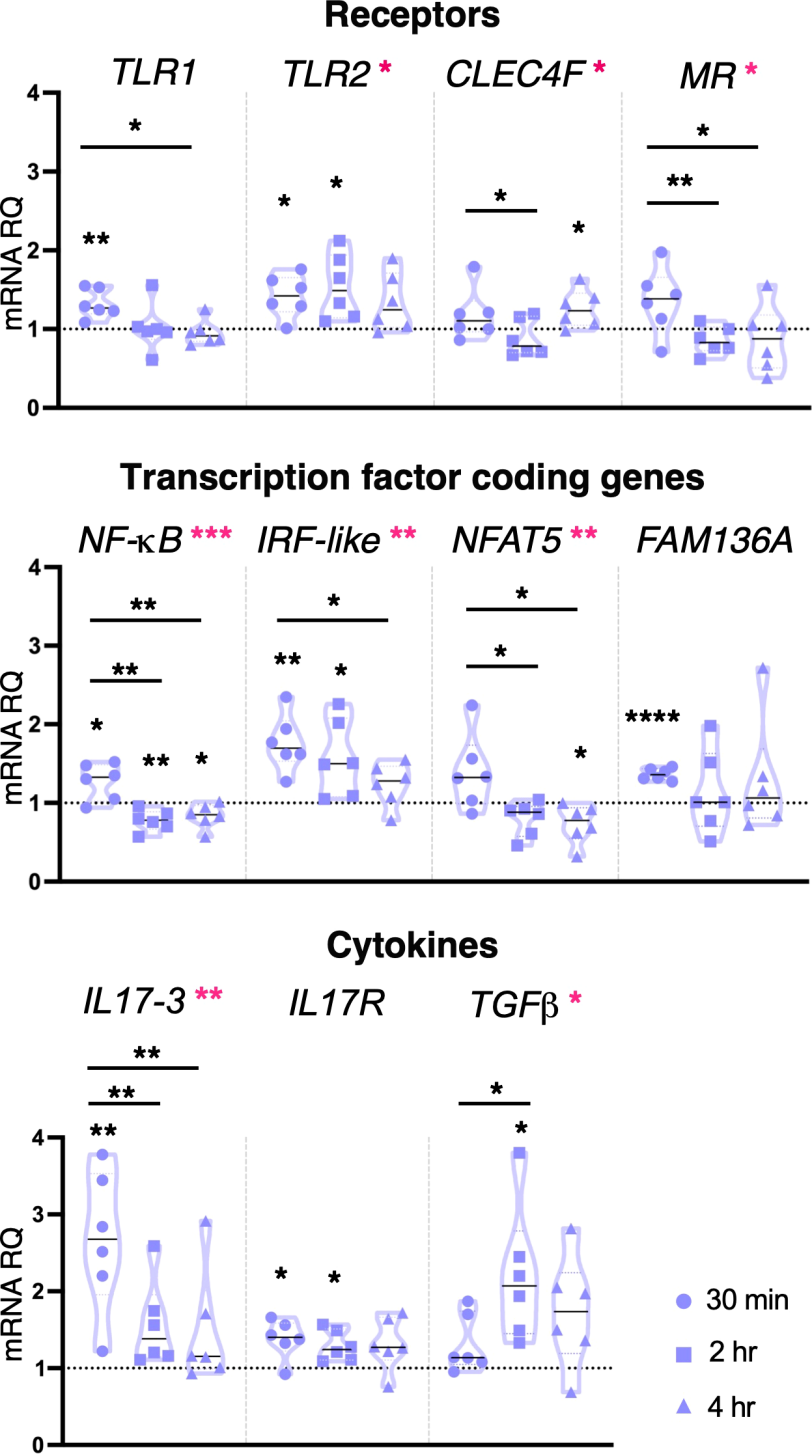
Effect of 1 μg/ml Pam2CSK4 treatment on gene expression. *C. robusta* juveniles at stage 4 of metamorphosis, treated with Pam2CSK4 at the concentration of 1 μg/ml, show significant changes in gene expression of *TLR1*, *TLR2*, *CLEC4F*, *MR*, *NF-κB*, *IRF-like*, *NFAT5*, *FAM136A*, *IL17-3*, *IL17R* and *TGFβ*, detected by RT-qPCR, after 30 min, 2 hr and 4 hr of treatment. Truncated violin plots represent the distribution and the density of numerical data of gene expression reported as fold changes (2^-ΔΔCt^) of mRNA Relative Quantification (mRNA RQ) compared to the corresponding control samples of juveniles (not treated) and reported as dotted black line. The black lines in violin plots indicate the median of data set (n = 6, biological replicates). Graphics shows also significant expression changes between two different time points of the treatment, indicated by horizontal black lines. Statistical methods: paired samples t-test, significance indicated by black asterisks; one-way ANOVA test, significance indicated by magenta asterisks. (*p. value < 0.05, **p. value < 0.01, ***p. value < 0.001, and ****p. value < 0.0001).

Unlike LPS, the inflammatory stimulus Pam2CSK4 was seen to induce transcriptional modulation of several molecules and pathways at all concentrations and time points. Following 1 μg/ml Pam2CSK4 treatment, *TLR1* was upregulated after 30 min treatment, *TLR2* after 30 min and 2 hr, and *CLEC4F* receptor after 4 hr treatment (**Figure 3**). While the expression of the genes encoding the receptor, *CLEC4M*, the Ig-domain containing proteins *FAM187A*, *FN*, *FN-like* and *TYRO3*, the cofactor *SYK*, was not regulated by Pam2CSK4 exposure, the expression of *FN*, *FN-like* and *TYRO3* was significantly modulated between 30 min and 4 hr treatments (**Supplementary Figure 2**). The expression of the transcription factor coding gene *NF-κB* was affected at all time points analyzed, with upregulation at 30 min and down-regulation at 2 hr and 4 hr; *IRF-like* was upregulated at both 30 min and 2 hr, whereas *NFAT5* was downregulated at 4 hr (**Figure 3**). Significant upregulation of *FAM136A* was observed after 30 min exposure (**Figure 3**). Concerning cytokines, *IL17-3* and *TGFβ* were significantly upregulated at 30 min and 2 hr treatment, respectively (**Figure 3**). Upregulation of *IL17R* was also observed at both 30 min and 2 hr time points (**Figure 3**). Conversely, cytokines *IL17-1*, *IL17-*2, *TNFα* and *MIF*, defensin *mamA*, and molecules of the complement system *C3* and *C3aR* were not affected in their transcriptional levels (**Supplementary Figure 2**). Statistical analysis by using one-way ANOVA test revealed that 1 μg/ml Pam2CSK4 had a significant effect on the modulation of genes coding for receptors (*TLR2*, *CLEC4F* and *MR*), transcription factors (*NF-κB*, *IRF-like* and *NFAT5*) and cytokines (*IL17-3* and *TGFβ*) (**Figure 3**).

**Figure 4:**
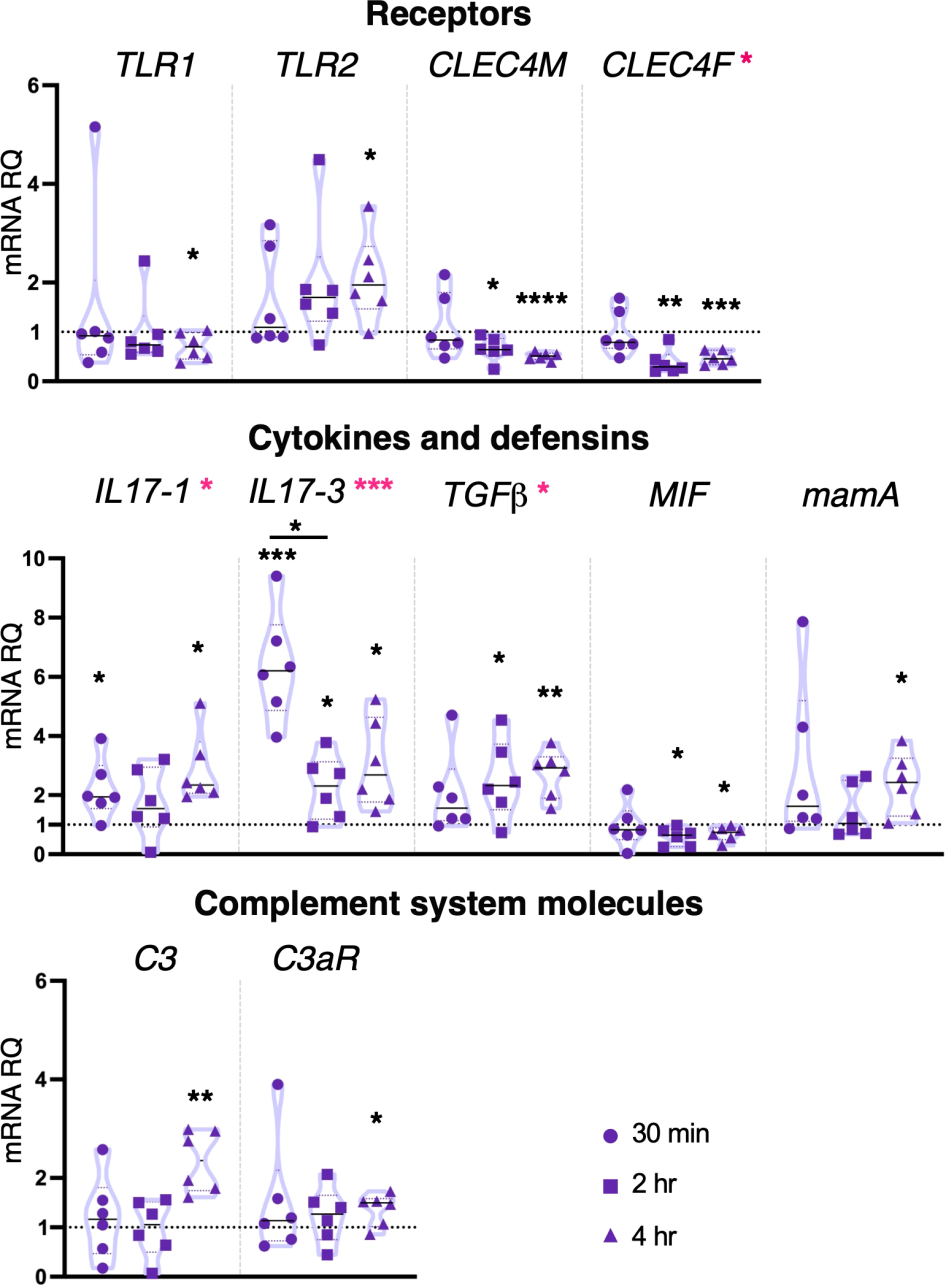
Effect of 10 μg/ml Pam2CSK4 treatment on gene expression. *C. robusta* juveniles at stage 4 of metamorphosis, treated with Pam2CSK4 at the concentration of 10 μg/ml, show significant changes in gene expression, detected by RT-qPCR, of *TLR1*, *TLR2*, *CLEC4M*, *CLEC4F*, *IL17-1*, *IL17-3*, *TGFβ*, *MIF*, *mamA*, *C3* and *C3aR*, after 30 min, 2 hr and 4 hr. Truncated violin plots represent the distribution and the density of numerical data of gene expression reported as fold changes (2^-ΔΔCt^) of mRNA Relative Quantification (mRNA RQ) compared to the corresponding control samples of juveniles (not treated) and reported as dotted black line. The black lines in each violin plots indicate the median of data set (n = 6, biological replicates). Graphics shows also significant expression changes between two different treatment time points, indicated by black lines. Statistical methods: paired samples t-test, significance indicated by black asterisks; one-way ANOVA test, significance indicated by magenta asterisks. (*p. value < 0.05, **p. value < 0.01, ***p. value < 0.001 and ****p. value < 0.0001).

Pam2CSK4 at the concentration of 10 μg/ml induced downregulation of *CLEC4M* and *CLEC4F* after both 2 and 4 hr treatment. TLR receptor coding genes were only affected after 4 hr, with downregulation of *TLR1* and upregulation of *TLR2* (**Figure 4**). Cytokines were upregulated following 30 min and 4 hr treatment (*IL17-1*), at all-time points (*IL17-3*) and 2 and 4 hr (*TGFβ*), while a downregulation of *MIF* was observed at 2 and 4 hr treatment (**Figure 4**). The highest concentration of Pam2CSK4 also induced upregulation of both defensin *mamA* and molecules of the complement system *C3* and *C3aR* after 4 hr treatment (**Figure 4**). No change in gene expression was observed for *MR*, *FAM187A*, *FN*, *FN-like*, *TYRO3, SYK*, *NF-κB*, *IRF-like* and *NFAT5*, protein coding gene *FAM136A*, and cytokines *TNFα*, *IL17-2* and the receptor *IL17R* (**Supplementary Figure 3**). Statistical analysis by using one-way ANOVA test revealed that 10 μg/ml Pam2CSK4 induces a significant modulation in the expression of *CLEC4F*, *IL17-1*, *IL17-3* and *TGFβ* (**Figure 4**).

Taken together, these data show that Pam2CSK4 is able to trigger the activation of both PRR pathways investigated, modulating the transcription of receptors (*TLRs* and *CLEC4M*) and transcription factors (*NF-κB*, *IRF-like* and *NFAT5*) with concentration-dependent timing of activation. The inflammatory state induced by this bacterial stimulus is demonstrated by the activation of pro-inflammatory cytokines like *IL17-1* and *IL17-3*. Finally, the upregulation of *TGFβ* cytokine at late time point indicates a resolution of the inflammatory response.

#### 3.2.3 Zymosan: effects on genes encoding for CLRs, Ig-containing molecules, cofactors, transcriptional factors, and cytokines

**Figure 5:**
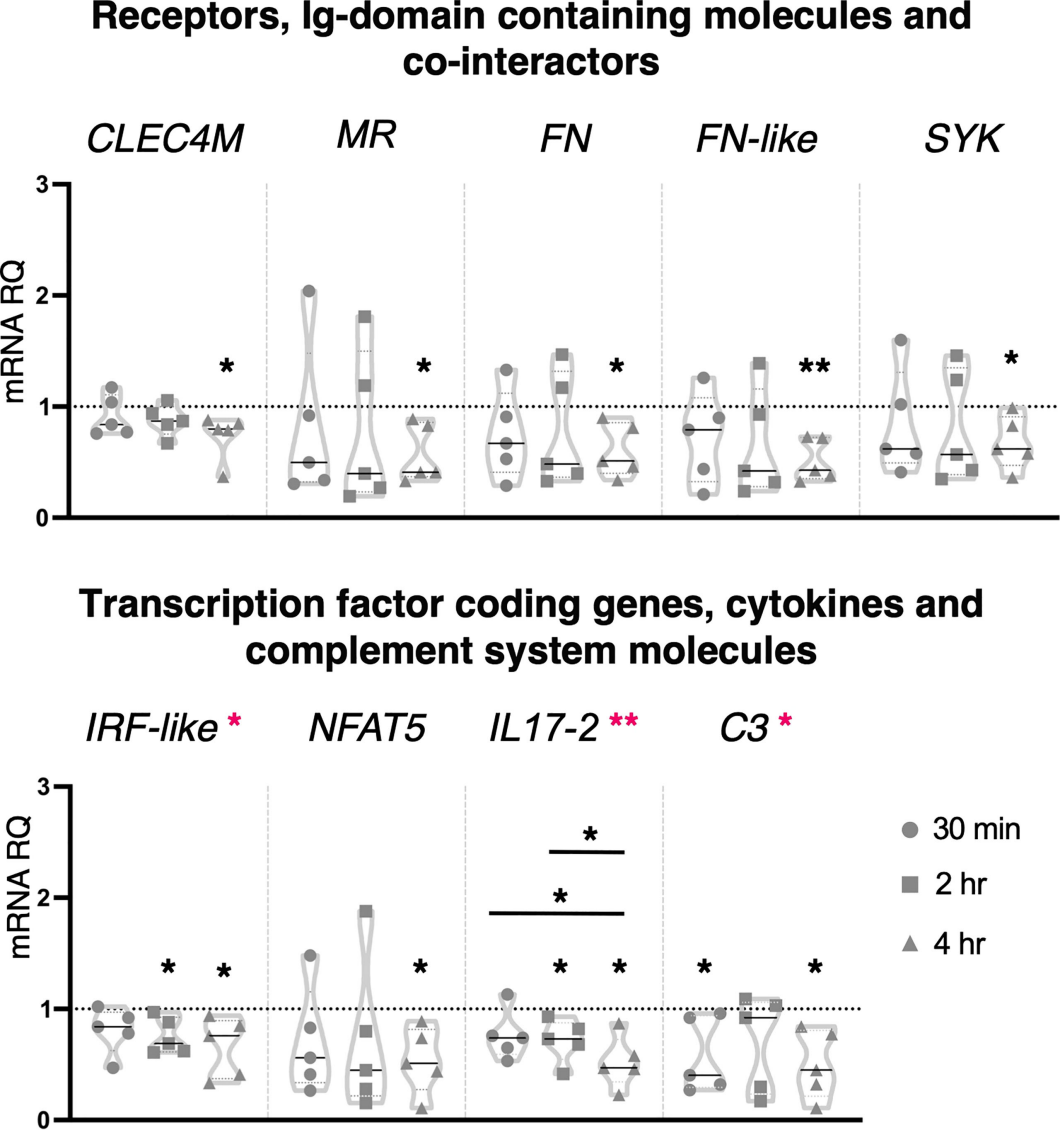
Effect of 10 μg/ml zymosan treatment on gene expression. *C. robusta* juveniles at stage 4 of metamorphosis, treated with zymosan at the concentration of 10 μg/ml, show significant changes in gene expression, detected by RT-qPCR, of *CLEC4M*, *MR*, *FN*, *FN-like*, *SYK*, *IRF-like*, *NFAT5*, *IL17-2* and *C3* after 30 min, 2 hr and 4 hr. Truncated violin plots represent the distribution and the density of numerical data of gene expression reported as fold changes (2^-ΔΔCt^) of mRNA Relative Quantification (mRNA RQ) compared to the corresponding control samples of juveniles (not treated) and reported as dotted black line. The black lines in each violin plots indicate the median of data set (n = 5, biological replicates). Graphics shows also significant expression changes between two different treatment time points, indicated by horizontal black lines. Statistical methods: paired samples t-test, significance indicated by black asterisks; one-way ANOVA test, significance indicated by magenta asterisks. (*p. value < 0.05, **p. value < 0.01).

*Ciona* juveniles exposed for 4 hr to the lower concentration of zymosan, 10 μg/ml, showed a significant downregulation of the receptors *CLEC4M*, *MR*, *FN*, *FN-like* and the cofactor *SYK* (**Figure 5**). A similar effect was observed on the transcription factor coding genes *NFAT5* and *IRF-like*, the last one being downregulated also at 2 hr (**Figure 5**). Downregulation of the cytokine *IL17-2* (2 and 4 hr) and of the complement component *C3* (30 min and 4 hr) was also observed (**Figure 5**). The other genes analyzed, such as receptors (*TLR1*, *TLR2*, *CLEC4F*), Ig domain-containing proteins (*FAM187A* and *TYRO3*), transcription factor coding genes (*NF-κB*), *FAM136A*, cytokines (*IL17-1*, *IL17-3*, *IL17R*, *TGFβ*, *TNFα* and *MIF*), defensins (*mamA*) and complement system components (*C3aR*) were not affected by this inflammatory stimulus (**Supplementary Figure 4**). One-way ANOVA analysis revealed a modulation in *IRF-like*, *IL17-2* and *C3* expression (**Figure 5**).

**Figure 6:**
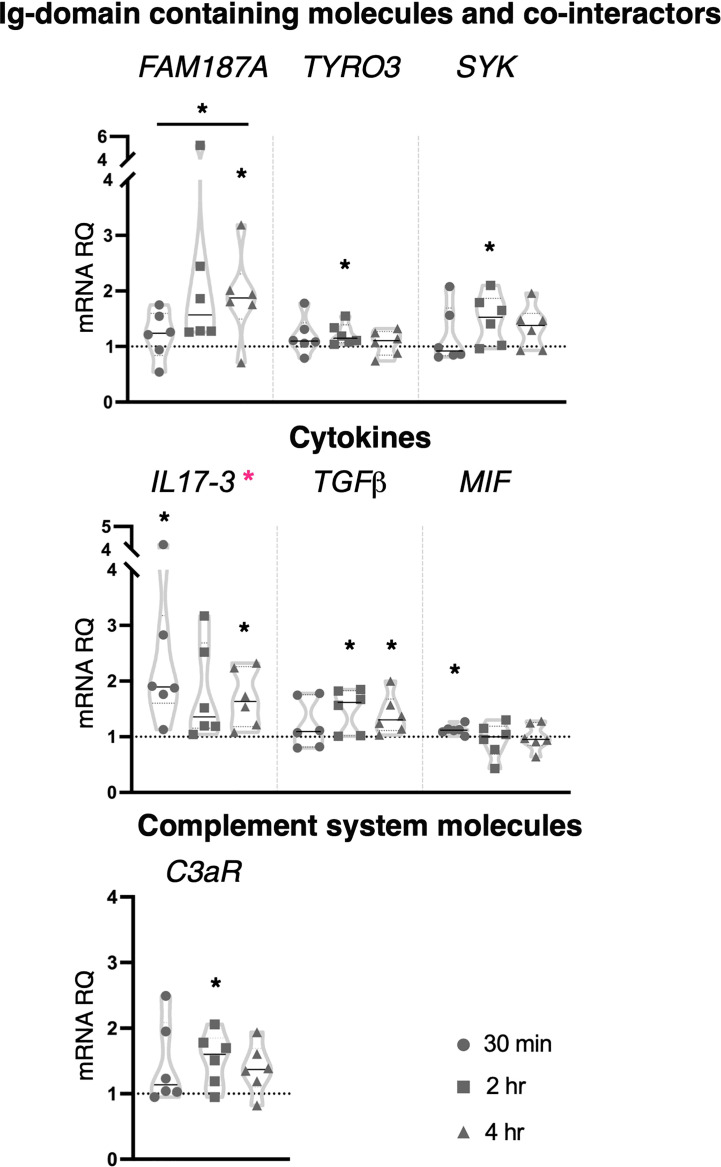
Effect of 100 μg/ml zymosan treatment on gene expression. *C. robusta* juveniles at stage 4 of metamorphosis, treated with zymosan at the concentration of 100 μg/ml, show significant changes in gene expression, detected by RT-qPCR, of *FAM187A*, *TYRO3*, *SYK*, *IL17-3*, *TGFβ*, *MIF* and *C3aR* after 30 min, 2 hr and 4 hr. Truncated violin plots represent the distribution and the density of numerical data of gene expression reported as fold changes (2^-ΔΔCt^) of mRNA Relative Quantification (mRNA RQ) compared to the corresponding control samples of juveniles (not treated) and reported as dotted black line. The black lines in each violin plots indicate the median of data set (n = 6, biological replicates). Graphics shows also significant expression changes between two different time points of the treatment, indicated by horizontal black lines. Statistical methods: paired samples t-test, significance indicated by black asterisks; one-way ANOVA test, significance indicated by magenta asterisks. (*p. value < 0.05).

Zymosan at the concentration of 100 μg/ml induced significant upregulation in the expression of genes coding for the Ig-domain containing proteins *FAM187A* (4 hr) and *TYRO3* (2 hr), cofactor *SYK* (2 hr), cytokines *IL17-3* (30 min and 4 hr), *TGFβ* (2 and 4 hr), *MIF* (30 min) and complement component *C3aR* (2 hr) (**Figure 6**). No effect was observed on the expression of the genes coding for receptors and Ig-domain containing proteins (*TLRs*, *CLE4M*, *CLEC4F*, *MR*, *FN* and *FN-like*), transcription factors (*NF-κB*, *IRF-like* and *NFAT5*), *FAM136A*, cytokines (*IL17-1*, *IL17-2*, *IL17R, TNFα*), defensins (*mamA*) and complement system molecules (*C3*) (**Supplementary Figure 5**). One-way ANOVA analysis showed a significant modulation of *IL17-3* expression during the analyzed time points, and a significant shift in the transcriptional response of *FAM187A* between 30 min and 4 hr treatments (**Figure 6**).

Collectively, the immune response induced by zymosan involves CLRs (*CLEC4M* and *MR*), cofactors (*SYK*) and transcription factors (*IRF-like* and *NFAT5*), suggesting an interplay of distinct PRR pathways. Among immune stimuli tested in this study, zymosan was the only one that affected the expression of Ig-containing molecules (*FAM187A*, *FN*, *FN-like* and *TYRO3*). Moreover, the activation of the inflammatory state following exposure to the higher concentration is highlighted by the modulation of the cytokine’s transcripts, pro-inflammatory (*IL17-3*), and consequently anti-inflammatory (*TGFβ*), indicating again a resolution of the inflammation.

### 3.3 Targeted proteomics

**Figure 7:**
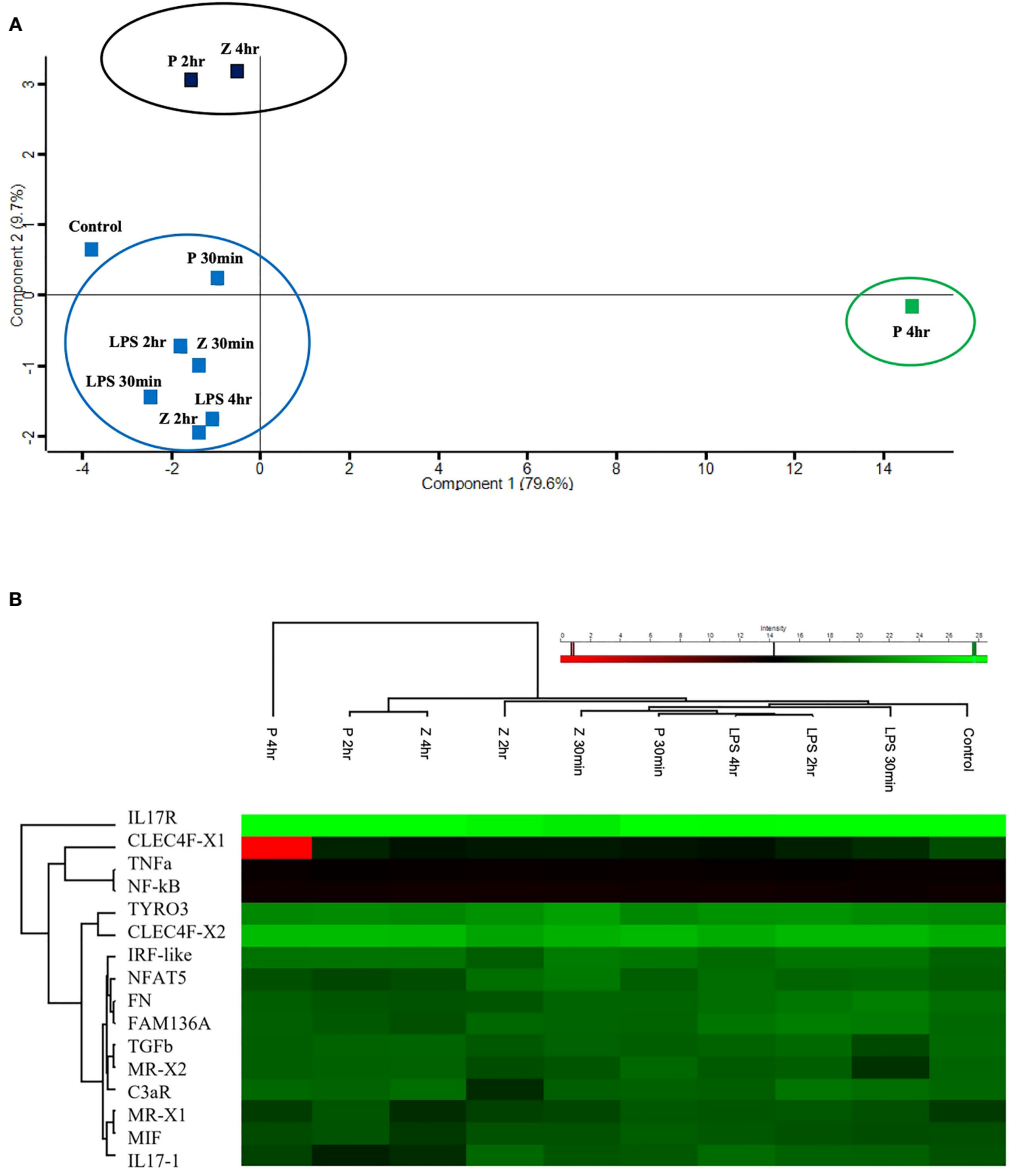
Targeted proteomic analysis of *C. robusta* juveniles expose to 10 μg/ml LPS, 1 μg/ml Pam2CSK4 and 100 μg/ml zymosan. **(A)** PCA analysis, clustering overall protein level variations at each time point of animals treated with microbial stimuli, shows similar protein level among control, LPS treated-, 2 hr and 4 hr zymosan treated-, and 30 min Pam2CSK4 treated- samples. 4 hr zymosan treated- and 2 hr Pam2CSK4 treated- samples have protein levels that cluster together and differs from control samples; whereas 4 hr Pam2CSK4 treated samples have protein level that highly differ from all the other treatment conditions. **(B)** Heatmap shows both hierarchical clustering of the treatment conditions and protein levels of the analyzed immune molecules, extracted from *C*. *robusta* juveniles expose to 10 μg/ml LPS, 1 μg/ml Pam2CSK4 and 100 μg/ml zymosan for 30 min, 2 hr and 4 hr and detected through LC-MRM/MS method. “P” and “Z” indicate Pam2CSK4 and zymosan treatments, respectively.

To explore the effect of each stimulus treatment at protein level, the targeted proteomic approach has been conceived for detecting changes in protein amount of the immune molecules here investigated. Indeed, A LC-MRM/MS method was developed for the detection of changes in protein abundance for the immune molecules. We first selected a panel of 1-3 proteotypic peptides for each protein and then recorded from 3 to 6 transitions (a combination of each precursor ion to several fragment ions) for each peptide. A total number of 32 peptides belonging to 16 proteins (listed in the **Supplementary Table 1** and in the **Figure 7B**) was selected by monitoring 139 transitions displaying a good instrumental response (the same retention time for all transitions and a peak area higher than 1,000). An example of MRM chromatogram was reported for one peptide of the NFAT5 protein (**Supplementary Figure 6**). After selecting the best instrumental response (see instrumental parameters in **Supplementary Table 2**), the relative quantification was performed by comparing the areas underlying the extracted ion chromatogram (EIC) peaks reflecting the differential expression of selected 16 proteins following the various stimulation *versus* the control sample. As an example, the EIC peak areas of two proteins, e.g. FAM136A and FN, were reported in a histogram representation (**Supplementary Figure 7**), displaying a similar trend in the samples. A significant downregulation was observed for the FAM136A and fibronectin protein along the Pam2CSK4 and zymosan treatment (independently from the stimulus kinetics) in comparison to the other samples, included the control.

The EIC peak areas were then uploaded on Perseus software to discover the response of *C. robusta* juveniles to stimulation with 10 μg/ml LPS, 1 μg/ml Pam2CSK4 and 100 μg/ml zymosan at 30 min, 2 hr and 4 hr of treatment compared to the control samples. The PCA analysis allowed to summarize and visualize the overall protein response to the microbial stimulation within a biplot where the control and treated samples were graphically represented. Among all revealed components, the first two (**Supplementary Table 3**) resulted in a two-dimensional PCA biplot (**Figure 7A**). PCA reduced the dimensionality of the multivariate data to two principal components explaining almost 90% variance (79.6% for Component 1 and 9.7% for Component 2) with minimal loss of information. The statistical analysis revealed a clear separation between a large cluster that included control, LPS (30 min, 2 hr, 4 hr), Pam2CSK4 (30 min) and zymosan (30 min, 2 hr) treatments, a small cluster consisting of 2 hr Pam2CSK4 and 4 hr zymosan treatments, and the 4 hr Pam2CSK4 treatment that was different from all other analyzed conditions (**Figure 7A**). This finding suggested that the stimulation of *C. robusta* juveniles with zymosan for 4 hr and Pam2CSK4 for 2 hr, and with Pam2CSK4 for 4hr induced a significant dysregulation of protein abundance as a consequence of microbial treatment response. Even the heatmap representation enabled the visualization of *hierarchical clustering* where the aforementioned microbial stimuli displayed the greatest response of immune molecules (**Figure 7B**).

### 3.4 Interactome of immune molecules

**Figure 8:**
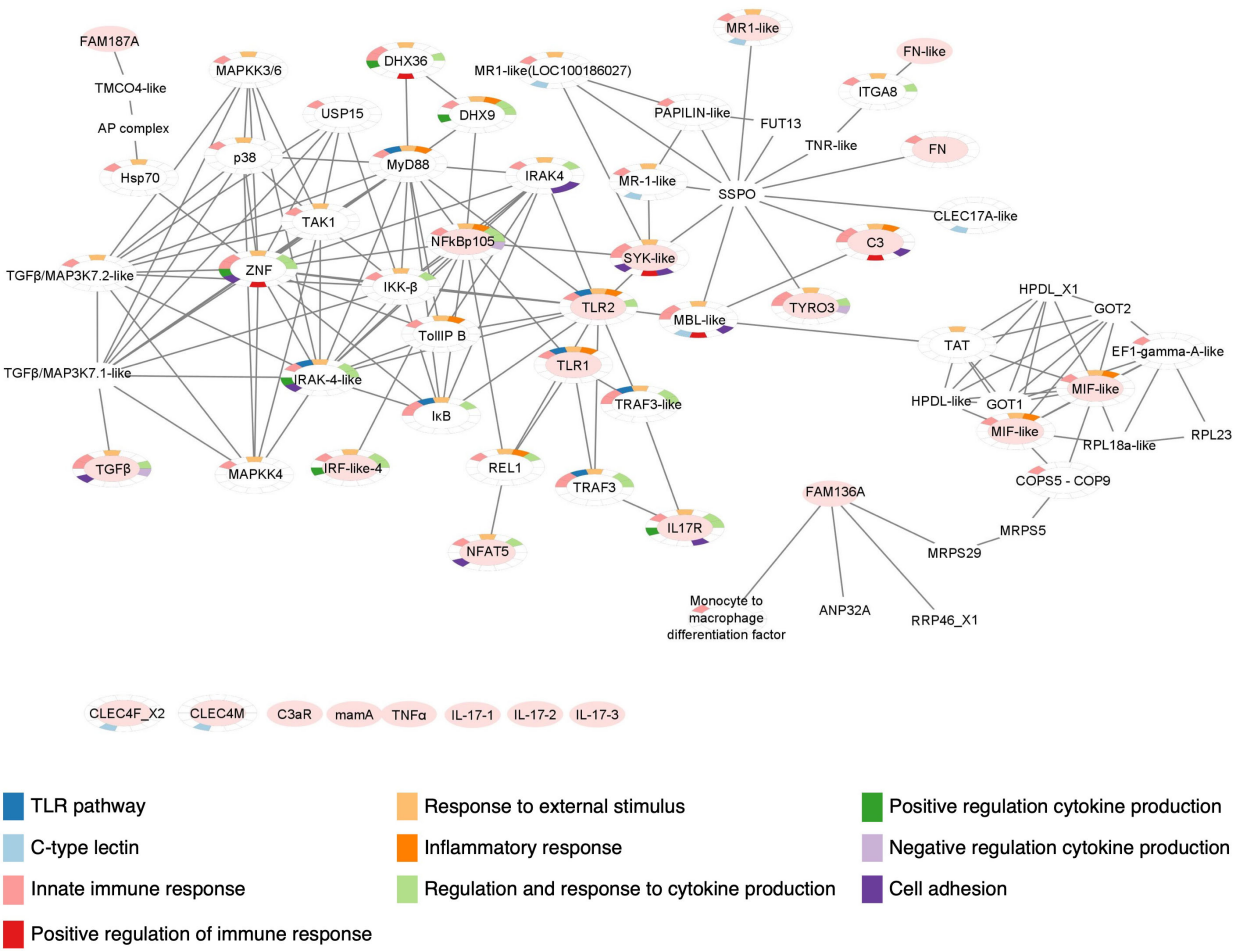
Protein-protein interaction network of *Ciona robusta* immune molecules. Protein-protein interaction map constructed with the STRING database and modified with Cytoscape, reveals the interaction between *C. robusta* immune molecules investigated through gene expression. Here, these molecules are indicated with an oval filled with light red color and those that are still not connected in the map are reported below. GO enrichment analysis is also indicated, highlighting in the color-legend some of the Biological Processes, related with immunological features, in which the molecules present in the map are involved.

A protein-protein interaction network, based on STRING output and then modified and merged in Cytoscape, has been constructed to include all the immune molecules whose gene expression has been investigated in this study (**Figure 8**). The interactome shows that both receptors TLR1 and TLR2 are connected with the three transcription factors NF-κB, IRF-like, NFAT5, and with the interleukin receptor IL17R. Notably, TLR2 is also connected with cofactor SYK, receptors MR and TYRO3, complement molecule C3, cytokine MIF and the molecule FAM136A. Although the lack of STRING annotations for IL17 gene products, possibly due to difficulties in investigating such proteins in C. *robusta*, the presence of connections with the receptor, IL17R, could provide clues about the interactions of these interleukins (**Figure 8**).

As reported for IL17s, lack of annotation information also affects other proteins, such as CLEC4M, CLEC4F, C3aR, mamA and TNFα. However, we found connection of similar domains belonging to other proteins, as in the case of CLEC17A-like that reveals a connection with the protein SYK (**Figure 8**). The interaction map shows that other main players of TLR signaling are connected, including MyD88, IRAK4, IKK, TAK1, p38, TRAF3 (13, 76, 77) and MAPKs. The genes coding for these proteins were not investigated in this work but should be future objects of similar studies. All the molecules included in the interaction map that were not investigated by transcriptional and proteomics analyses in this study, are listed in the **Supplementary Table 4**.

The enrichment analysis has been performed through STRING Enrichment app in Cytoscape to depict the Biological Processes, Molecular Functions and Cellular Components in which these molecules are involved. Some of the results are represented as Split Donut Chart (chosen option in the Network specific settings for STRING Enrichment table) in the protein-protein interaction network, using certain colors from the Enrichment color palette to underline specific outputs. To generate a map easier to understand, we have highlighted the biological processes that are more significant for this study. These comprise TLR signaling pathways (GO:0002224), innate immunity including inflammation and response to external stimulus (GO:0050896, GO:0002682, GO:0050794, GO:0050778), regulation and response to cytokine production (GO:0034097, GO:0001817, GO:0001818, GO:0001819), CTL or CRD domains (SM00034), and leukocyte-mediated immunity and adhesion to endothelial cells (GO:0045785, GO:0050900, GO:0002443) (**Figure 8**).

## 4 Discussion

The marine invertebrate *C. robusta* has recently become an excellent experimental organism for studying gut mucosal immunity, including processes mediating host mucosal-microbial associations (7, 8, 40, 78–82). In this work we used *Ciona* for investigating the inflammatory response activated by three PAMPs (LPS, Pam2CSK4 and zymosan) and for developing an invertebrate inflammatory model to use in research fields, from comparative immunology to translational biology and drug discovery.

### 4.1 Inflammation and the “patterns of pathogenesis” hypothesis

To interpret the inflammatory response in ascidian juveniles, it is important to recall some basic immunological concepts, such as the definition of inflammation and which factors/conditions activate it. The term “inflammation” defines the process triggered by innate immune cells when the homeostatic state is altered due to microbial infection or tissue injury (11–13). Marine organisms are continuously exposed to, and challenged by, a multitude of microorganisms (e.g., bacteria, archaea, fungi, viruses, protozoans) inhabiting the surrounding environment (83). These microbes may be beneficial for the host by helping to shape the immune system and influencing developmental and physiological processes (84). The superorganism theory emphasizes how the concept of *self* and *non-self* has changed over time by incorporating host microbiota in the definition of *self* (84–86). During early development, the crosstalk between host and microbes is crucial to shape immune system maturation, that will allow to discriminate between *self* and *non-self*, and to establish a homeostatic state with beneficial components (86). These processes are mostly studied within the gastrointestinal tract where the symbiotic interactions mostly take place (87, 88). This equilibrium is broken when pathogenic microbes, or their components (as PAMPs), invade the host epithelial barrier and induce infection. Colonization and invasion by pathogens activate an inflammatory response that primarily involves PRRs (and their signaling pathways), the main players of the innate immune system (13, 68, 89, 90), with the aim to eliminate the infectious agents and to restore homeostasis (11, 12). PRRs have broad specificity and can recognize many PAMPs, which have a common structural motifs or patterns, thus representing a sort of pathogenicity markers (89, 91). In 2009, Vance and coauthors have proposed the “patterns of pathogenesis”, or POP hypothesis, according to which the immune system recognizes pathogens not only by virtue of the presence of PAMPs but also by their pathogenic behaviors (92). These include growth upon host invasion, cytosolic invasion and disruption of the normal functions in the host cell cytoskeleton (92). On these grounds, a microbe can be considered pathogenic or nonpathogenic depending on the site of infection and on the immune state of the host. Hence, POP do not define a pathogen, rather a pathogenic behavior (86, 92). These new concepts may help to better understand the effect of PAMPs on the immune response, and thus on the onset of the inflammation, in different organisms and physiological states.

Based on the POP hypothesis, it is important to consider the physiological conditions at which the immune challenge is encountered. *Ciona* stage 4 juveniles are immunological naïve and probably they still do not have a stable microbiota. As they start interacting with the surrounding environment by seawater filtration, ascidian juveniles are here exposed to resuspended microbial components (8, 33, 40) and an immune response is observed if microbial components interact with the epithelial barrier. In line with *Ciona* LPS injection-based studies (14, 25, 93), the resuspension approach used herein may act differently in terms of immune activation patterns on older juveniles, like stage 8 (2^nd^ ascidian stage) (33).

### 4.2 Stimulus-specific response of innate immunity in *C. robusta* juveniles

In this work we found that LPS, the most common inflammatory stimulus used in vertebrates (13, 68, 94, 95), ascidians and other marine invertebrates (96–99), does not significantly alter gene expression in the adopted experimental setup, that are naïve metamorphic stage 4 juveniles. In line with the transcriptional results, targeted proteomics data show that LPS treatment does not elicit protein level variations. The POP hypothesis may help to explain the lack of an immune response to LPS in *C. robusta* juveniles treated by LPS resuspension. Since these organisms inhabit a habitat rich in LPS-containing Gram-negative bacteria (78) that are continuously filtered by the organism and interact with host mucosal sites, ascidian juveniles may not recognize LPS as a PAMP and/or LPS may not cross the epithelial barrier of their gastrointestinal tract. Instead, the other two microbial components that we used as inflammatory stimuli, the bacterial Pam2CSK4 and the fungal zymosan, are apparently able to interact with the epithelial barrier and then be sensed by host immune surveillance as PAMPs, thus triggering an immune response.

Pam2CSK4, which is not commonly used as a microbial stimulus in vertebrates as well as marine organisms, has been shown to bind the heterodimer TLR1/TLR6 (38) and, in monocytes, to enhance expression and function of Fcγ, a receptor involved in phagocytosis and inflammatory cytokine production (100). In mice, *in vitro* and *in vivo* stimulation of macrophages induces the activation of MAP kinase and NK-κB pathways upon TLR2 binding (101). Also, Pam2CSK4 treatment of human platelets *in vitro* activates TLR2/TLR6 complex and initiates signaling events that stimulate increase of NF-kB protein level and interactions between platelets and endothelial cells (ECs). This event increases inflammatory cytokine production and reduces EC permeability (102). In our experiments, Pam2CSK4 was the most effective inflammatory stimulus in ascidian juveniles, featuring a concentration-dependent influence on both TLR and Dectin-1 pathways. Also, it is worth mentioning that 1 μg/ml is lower than the concentration of Pam2CSK4 used on human cell lines (10 ug/ml) (100, 102), suggesting that ascidian juveniles are highly responsive to this microbial stimulus. The immune response to Pam2CSK4 observed in *Ciona* juveniles at stage 4 include the modulation of the PRRs expression, TLRs and CLECs, and of the transcription factors just at the lower concentration tested. While expression data show that Pam2CSK4 exposure modulates both TLR and Dectin-1 pathways, the protein-protein interaction network could not confirm it, as long as CLEC4M and CLEC4F receptors are concerned.

The time of activation of each pathway depends on the amount of microbial component that interacts with host mucosal barrier. We may hypothesize a stronger, but vital, immune response induced by 10 μg/ml Pam2CSK4 that can penetrate mucus barrier (due to the higher amount of Pam2CSK4 molecules), interact with epithelial layer and affect gene expression of *Ciona* complement system components C3 and C3aR, that consequently can activate cell adhesion, chemotaxis and phagocytosis processes in order to fight the invading microbial molecules (103, 104). Moreover, as in human platelets (102), Pam2CSK4 is able to alter transcriptional levels of cytokines (e.g., *IL17-3* and *TGFβ*) in *Ciona*. The activation of *TGFβ* following *IL17-3* modulation could be explained as a possible resolution of the inflammation induced by this microbial stimulus (105), in agreement with the observation that juveniles after 24 hr treatment are healthy (**Figure 1B**). That 10 μg/ml Pam2CSK4 can induce a strong inflammatory response is corroborated by the evidence of expression changes of other cytokine coding genes like *IL17-1* and *MIF*. In mammals, *MIF* has pro-inflammatory and immunoregulatory properties, and upregulates *TLR4* expression (106). In LPS-injected *Ciona* adults, a major role of MIF signaling pathway has been described in regulating *IL17s* and TGF*β* expression (93). A similar effect is observed also here in the inflammatory state induced by Pam2CSK4 treatment, where a downregulation of *MIF* expression is concurrent with the upregulation of *IL17s* and *TGFβ* (after 2 and 4 hr treatment with 10 μg/ml Pam2CSK4).

**Figure 9:**
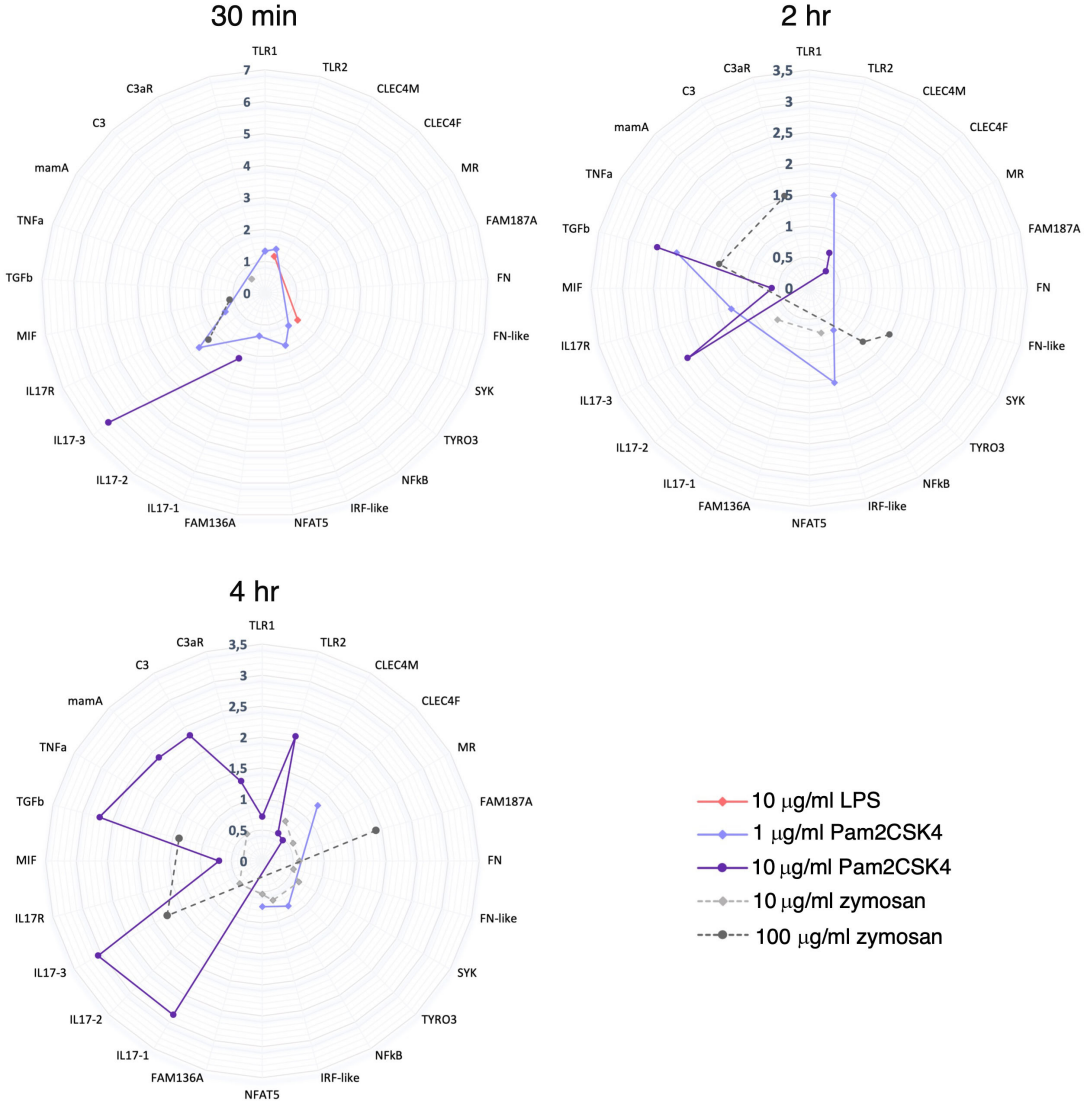
Summary of gene expression modulation at 30 min, 2 hr and 4 hr treatment. Radar plots show a summary of the genes that are (significantly) modulated at each time point treatment (30 min, 2 hr and 4 hr) by 10 μg/ml LPS, 1 and 10 μg/ml Pam2CSK4 and 10 and 100 μg/ml zymosan. 10 μg/ml LPS modulated just 2 genes at 30 min treatment. 1 μg/ml Pam2CSK4 modulate a higher number of genes at 30 min (7 genes) and 2 hr (4 genes) respect to 4 hr (3 genes) treatment. On the contrary, 10 μg/ml Pam2CSK4 has a major effect at late time points, 2 hr (5 genes) and 4 hr (11 genes), respect to the 30 min (2 genes) treatment. zymosan treatment, at both concentrations used, has an effect at the late time points, 2 hr (10 μg/ml, 2 genes; 100 μg/ml, 4 genes) and 4 hr (10 μg/ml, 9 genes; 100μg/ml, 3 genes) respect to the 30 min treatment (10 μg/ml, 1 genes; 100μg/ml, 2 genes).

Compared to Pam2CSK4 infection, zymosan modulates a smaller and diverse set of molecules and pathways in *Ciona* juveniles (**Figure 9**). As a cell wall preparation of yeast *S. cerevisiae*, zymosan is a mix of glucans, mannans, mannoproteins and chitin (107). These components are implicated in yeast recognition by innate immune cells, stimulating phagocytosis by macrophages (108) and cytokine production (i.e. TNFα, IL1β, IL-10 and TGFβ) by monocytes, macrophages and dendritic cells (39, 109–111). In vertebrates, zymosan activates both TLR and Dectin-1 pathways (39). Moreover, a cell signaling cascade activated by the binding of β-glucan to Dectin-1 receptor can initiate both Syk-dependent and Syk-independent cascades (68, 112). In mammals, Syk is involved in cytokine transcription upon recruitment by Dectin-1 (113). However, Syk factor is involved in both PRR pathways (68, 114).

In *Ciona*, the protein-protein interaction network here generated highlights a potential connection among TLR2, SYK and MR molecules. Although activation of *Ciona* TLRs was induced by zymosan in a heterologous cellular system (17), here we did not observe changes in the expression of the two *TLR* genes investigated, but we found an alteration of *SYK* expression that does not permit us to rule out the hypothesis of an involvement of TLR pathway. The high zymosan concentration seems to induce a stronger inflammatory response that affect mainly gene transcription of cofactors involved in both TLR and Dectin-1 pathways, highlighting again an interconnection between the two pathways as observed in juveniles exposed to Pam2CSK4, although we did not observe a direct effect on the expression of the PRRs investigated. The finding that high concentration induces upregulation of the gene coding for TYRO3, a coreceptor involved in the resolution of inflammation, is in line with the upregulation of *TGFβ* observed after the increase of the expression of the two pro-inflammatory cytokines *IL17-3* and *MIF*. The role of TYRO3 in the negative regulation of cytokine production has been depicted also by GO analysis of the biological process of *Ciona* protein-protein interaction map.

In this study, we have observed an effect on the transcriptional levels of Ig-domain containing molecules (*FAM187A*, *FN* and *FN-like*) at both zymosan concentrations. Of note, the connection of these molecules with the Dectin-1 and TLR immune pathways was confirmed by the protein-protein interaction network. The immune role of these Ig-domain containing molecules represents an interesting starting point for future investigation in deciphering their role in the inflammatory response to fungal wall components. Moreover, the finding that *Ciona FAM187A* is phylogenetically related to *B. schlosseri TREML2* gene and that homology search analysis of *C. robusta* genome and proteome revealed the presence of genes coding for SYK (a downstream effector of the TREM2 pathway) and TYRO3, the latter binding in mammals TYRO protein tyrosine kinase binding protein (TYROBP), also known as DNAX-activating protein of 12 kDa (DAP12) whose putative receptor is TREM2 (115), induce to hypothesize that the existence of the TREM2 signaling is an ancient trait whose origin dates back to the common chordate ancestor. It also prompts for a better understanding of the evolution of this pathway in immune function, and in particular in the response to molecules of fungal origin.

Finally, we report that zymosan affect the transcription of the *Ciona* complement system, suggesting the activation of a phagocytosis process. This has been observed also in mammals, where zymosan is phagocytosed by macrophages with or without opsonization and can activate alternative pathway of complement system (116–118).

### 4.3 Targeted proteomics supports transcriptional data and show time delay between mRNA and protein expression

Proteomic data reveal an effect on protein level modulation after 2 hr Pam2CSK4 and 4 hr zymosan treatments, and a major effect after 4 hr treatment with Pam2CSK4. Here, the targeted proteomic analysis performed on a small subset of proteins (encoded by the genes analyzed at the transcriptional level) and experimental conditions highlights that *i*) Pam2CSK4 has a major effect in the immune regulation respect to zymosan, and that *ii*) the immune response to these two PAMPs differs in the time of activation. As to the latter aspect, low concentration of Pam2CSK4 is sufficient to induce early expression changes (30 min and 2 hr) as suggested by the transcriptional modulation of a higher number of genes at these time points (**Figure 9**). This evidence corresponds to a significant effect in a general protein modulation at 2 hr and 4 hr of treatment (**Figure 7**). A similar delay is observed also in the case of zymosan, which induces a significant modulation of protein levels only at 4 hr, compared to the transcriptional response observed at 2 and 4 hr (**Figures 7**, **9**). These analyses help draw a first consideration concerning the temporal delay observed in the synthesis of proteins with respect to mRNA expression. The initiation of protein translation occurs within minutes after mRNA export into the cytoplasm, thus justifying the lag between transcription and translation. However, we cannot exclude the involvement of mechanisms, such as translational control through RNA binding proteins, that tightly regulate the production of specific proteins, thereby helping to resolve the inflammatory response (119). In mouse dendritic cells treated with LPS, time delay between transcriptional induction and protein level increases was described, with rapid expression of immune response genes (5 hr post LPS treatment) followed by the best quantitative correlation of protein levels to the mRNA levels at 12 hr (120).

### 4.4 Concluding remarks

In our study, we have added a further tile in the use of the ascidian *C. robusta* as an experimental system in comparative immunology field. Specifically, we have *i*) developed *Ciona* as an *in vivo* inflammatory model for studying the activation of the immune response to selected microbial stimuli, showing an interconnection between different PRR pathways and indicating the upregulation of cytokines gene expression (*IL17-3* and *TGFβ*) as markers of inflammation, *ii*) constructed a first protein-protein interaction map that can help to predict potential molecular interactions, and *iii*) correlated changes observed at transcriptional and translational levels. This new marine invertebrate inflammatory model represents the starting point for future studies, that include either large-scale sequencing or other “-omics” approaches for better defining the cellular pathways or biological processes affected by microbial treatments, but also for investigating host response to PAMPs in different physiological conditions and at different stages of maturation of the immune system. These advancements will contribute to our understanding of the crosstalk between host and microbiota and to test the POP hypothesis. As suggested by Newton and Dixit (2012), it is important to understand how, in a whole organism, innate immune cells exposed to multiple inflammatory stimuli can integrate signaling triggered by different receptors to identify critical components that can be targeted for therapeutic benefit in inflammatory disorder (13). In this framework we believe that, based on the results obtained in this study, this marine model organism could represent a proficient experimental system. Future studies can lead to the use of *C. robusta* experimental system in translation research or in any kind of approach (i.e., biotechnological or ecotoxicological) where the effect of molecules, either drugs or pollutants, on the activation and regulation of the innate immune system has to be investigated, like in large-scale screening of inflammatory modulators.

## Data availability statement

The original contributions presented in the study are included in the article/**Supplementary Material**. Further inquiries can be directed to the corresponding authors.

## Ethics statement

Ethical review and approval was not required for the study on animals in accordance with the local legislation and institutional requirements.

## Author contributions

AL, CP, RM, AS and PS contributed to conception and design of the study. AL and CP performed *in vivo* treatments and gene expression analysis. CP executed *in silico* analysis and wrote the corresponding sections of the manuscript. GP, AI and AA performed proteomic analysis, its data curation and wrote the corresponding sections of the manuscript. AL performed interpretation and curation of data and wrote the first full version of the manuscript. RM, AS and PS reviewed and edited the manuscript, and contributed to the discussion. AL and PS supervised the project. All authors contributed to the article and approved the submitted version.
