# Supplementary material for: Transcriptional and proteomic analysis of the innate immune response to microbial stimuli in a model invertebrate chordate
Source: Front Immunol. 2023 Aug 2;14:1217077. doi: 10.3389/fimmu.2023.1217077 (PMC10433773; doi:10.3389/fimmu.2023.1217077)
Supplement: Supplementary file 1 [file DataSheet_1.docx]

Supplementary Material

**Transcriptional and proteomic analysis of the innate immune response to microbial stimuli in a model invertebrate chordate**

**Assunta Liberti^1^**^†^***, Carla Pollastro^1^**^†^**^#^, Gabriella Pinto^2,3^, Anna Illiano^2,3^, Rita Marino^1^, Angela Amoresano^2,3^, Antonietta Spagnuolo^1^ and Paolo Sordino^4^***

^1^ Biology and Evolution of Marine Organisms (BEOM), Stazione Zoologica Anton Dohrn, Naples, Italy.

^2^ Department of Chemical Sciences, University of Naples Federico II, Via Cinthia 26, 80126 Naples, Italy.

^3^ Istituto Nazionale Biostrutture e Biosistemi-Consorzio Interuniversitario, Viale delle Medaglie d'Oro, 305, 00136 Rome, Italy.

^4^ Biology and Evolution of Marine Organisms (BEOM), Stazione Zoologica Anton Dohrn, Sicily Marine Centre, Messina, Italy.

^#^ Current address: TIGEM - Telethon Institute of Genetics and Medicine, 80078 Naples, IT.

^†^ Equal contribution.

*** Correspondence:**

Assunta Liberti [assunta.liberti@szn.it](mailto:assunta.liberti@szn.it), [assusy.liberti@gmail.com](mailto:assusy.liberti@gmail.com)

Paolo Sordino [paolo.sordino@szn.it](mailto:paolo.sordino@szn.it)


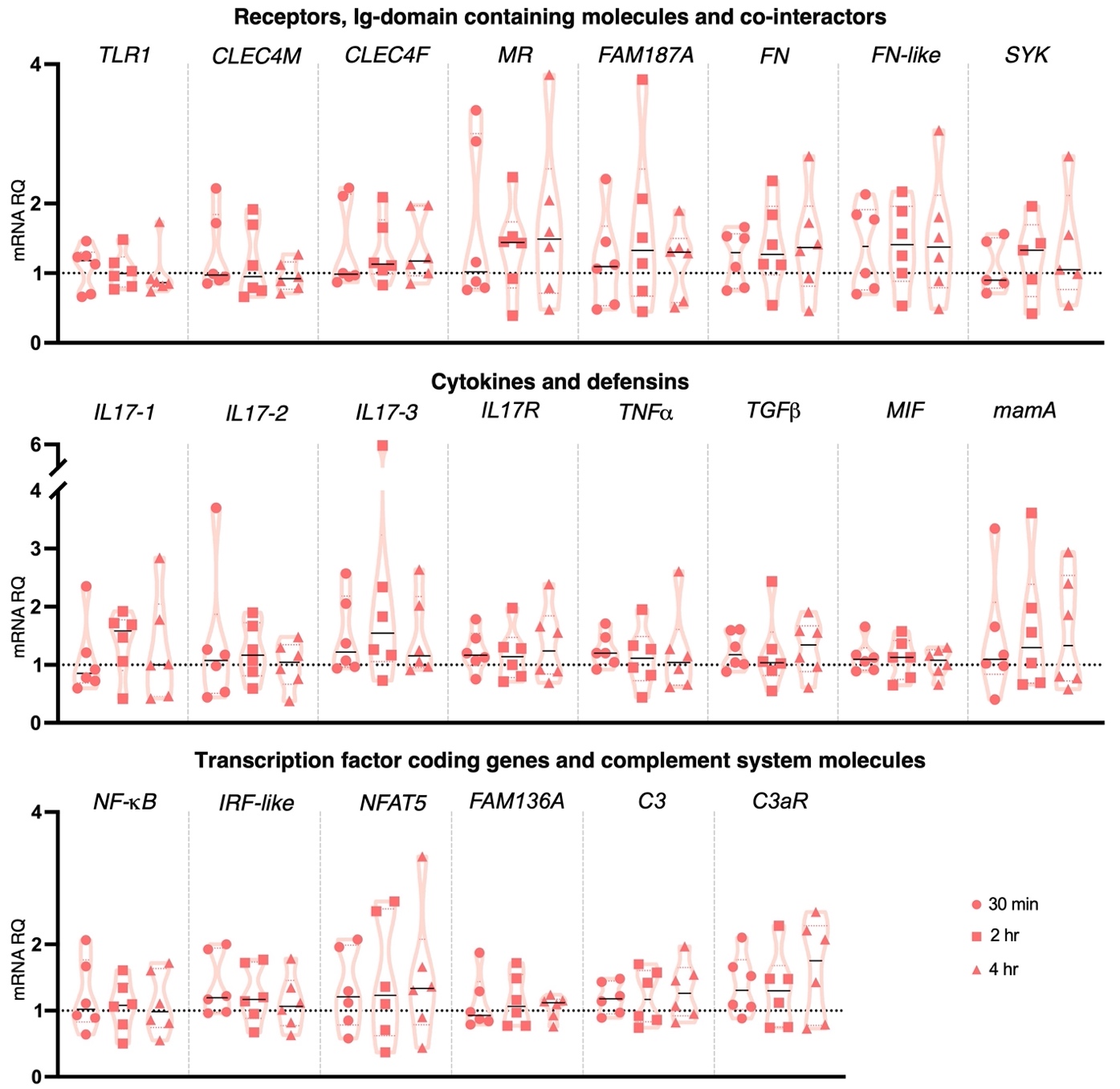


**Supplementary Figure 1: Genes not effected by treatment with 10 μg/ml LPS.** *C. robusta* juveniles treated with 10 μg/ml LPS for 30 min, 2 hr and 4 hr, do not show significant changes in the expression, measured by RT-qPCR, of most of the genes investigated, such as *TLR1*, *CLEC4M*, *CLEC4F*, *MR*, *FAM187A*, *FN*, *FN-like*, *SYK*, *IL17-1*, *IL17-2*, *IL17-3*, *IL17R*, *TNFα*, *TGFβ*, *MIF*, *mamA*, *NF-κB*, *IRF-like*, *NFAT5*, *FAM136A*, *C3* and *C3aR*. Truncated violin plots represent the distribution and the density of numerical data of gene expression reported as fold changes (2^-ΔΔCt^) of mRNA Relative Quantification (mRNA RQ) compared to the corresponding control samples of juveniles (not treated) and reported as dotted black line. The black lines in each violin plots indicate the median of data set (n = 6, biological replicates). Statistical methods: paired samples t-test and one-way ANOVA test.


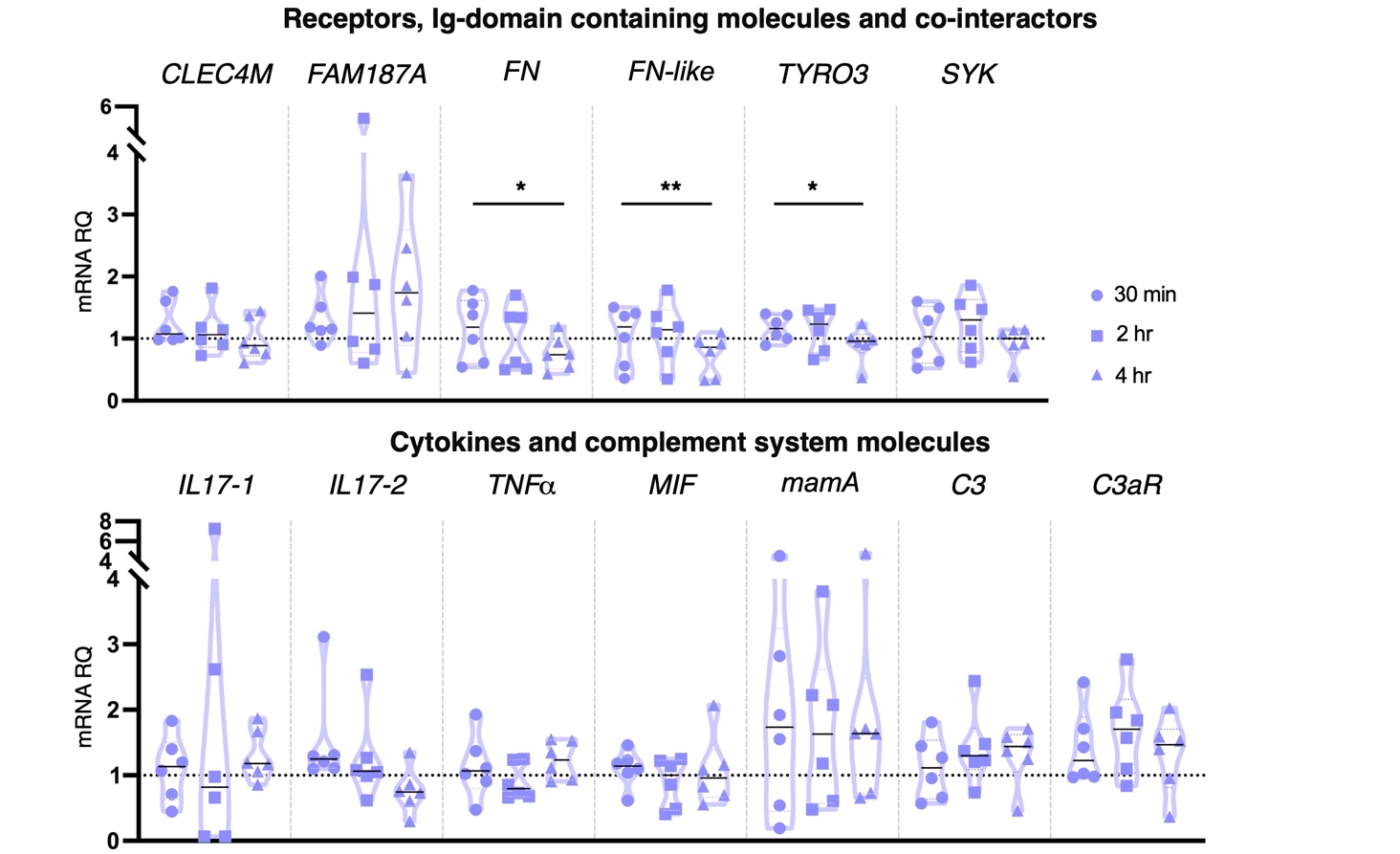


**Supplementary Figure 2: Genes not effected by treatment with 1 μg/ml Pam2CSK4.** *C. robusta* juveniles treated with 1 μg/ml Pam2CSK4 for 30 min, 2 hr and 4 hr, do not show significant changes in gene expression, measured by RT-qPCR, of *CLEC4M*, *FAM187A*, *FN*, *FN-like*, *TYRO3*, *SYK*, *IL17-1*, *IL17-2*, *TNFα*, *MIF*, *mamA*, *C3* and *C3aR*. Truncated violin plots represent the distribution and the density of numerical data of gene expression reported as fold changes (2^-ΔΔCt^) of mRNA Relative Quantification (mRNA RQ) compared to the corresponding control samples of juveniles (not treated) and reported as dotted black line. The black lines in each violin plots indicate the median of data set (n = 6, biological replicates). However, significance in gene expression changes is observed between two different time points of the treatment, indicated by horizontal black lines. Statistical methods: paired samples t-test (black asterisks) and one-way ANOVA test. (* p. value < 0.05; ** p. value < 0.01).


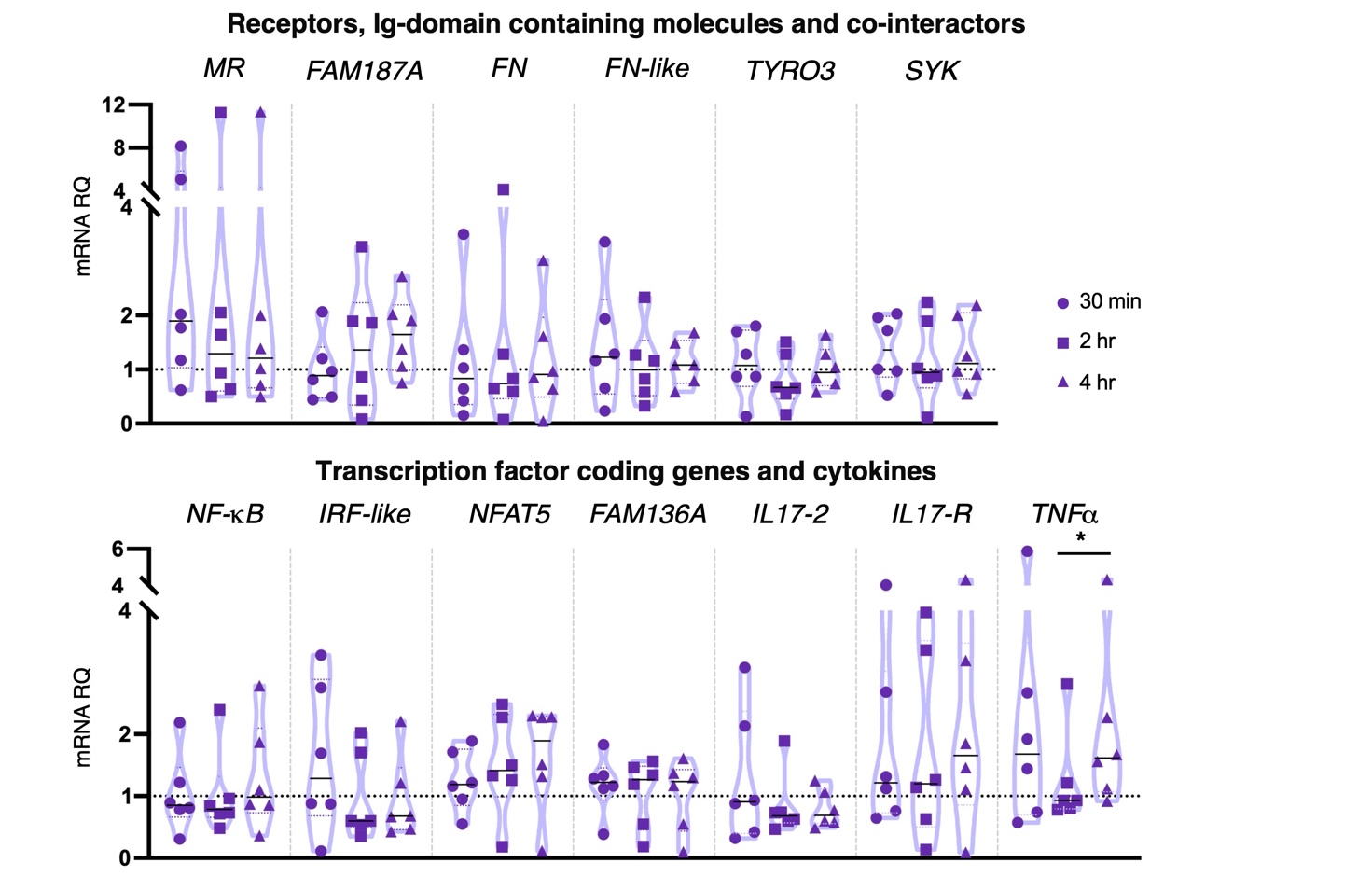


**Supplementary Figure 3: Genes not effected by treatment with 10 μg/ml Pam2CSK4.** *C. robusta* juveniles treated with 10 μg/ml Pam2CSK4 for 30 min, 2 hr and 4 hr, do not show significant changes in gene expression, measured by RT-qPCR, of *MR*, *FAM187A*, *FN*, *FN-like*, *TYRO3*, *SYK*, *NF-κB*, *IRF-like*, *NFAT5*, *FAM136A*, *IL17-2*, *IL17R* and *TNFα*. Truncated violin plots represent the distribution and the density of numerical data of gene expression reported as fold changes (2^-ΔΔCt^) of mRNA Relative Quantification (mRNA RQ) compared to the corresponding control samples of juveniles (not treated) and reported as dotted black line. The black lines in each violin plots indicate the median of data set (n = 6, biological replicates). However, significance in gene expression changes is observed between two different time points of the treatment, indicated by horizontal black lines. Statistical methods: paired samples t-test (black asterisk) and one-way ANOVA test. (* p. value < 0.05).


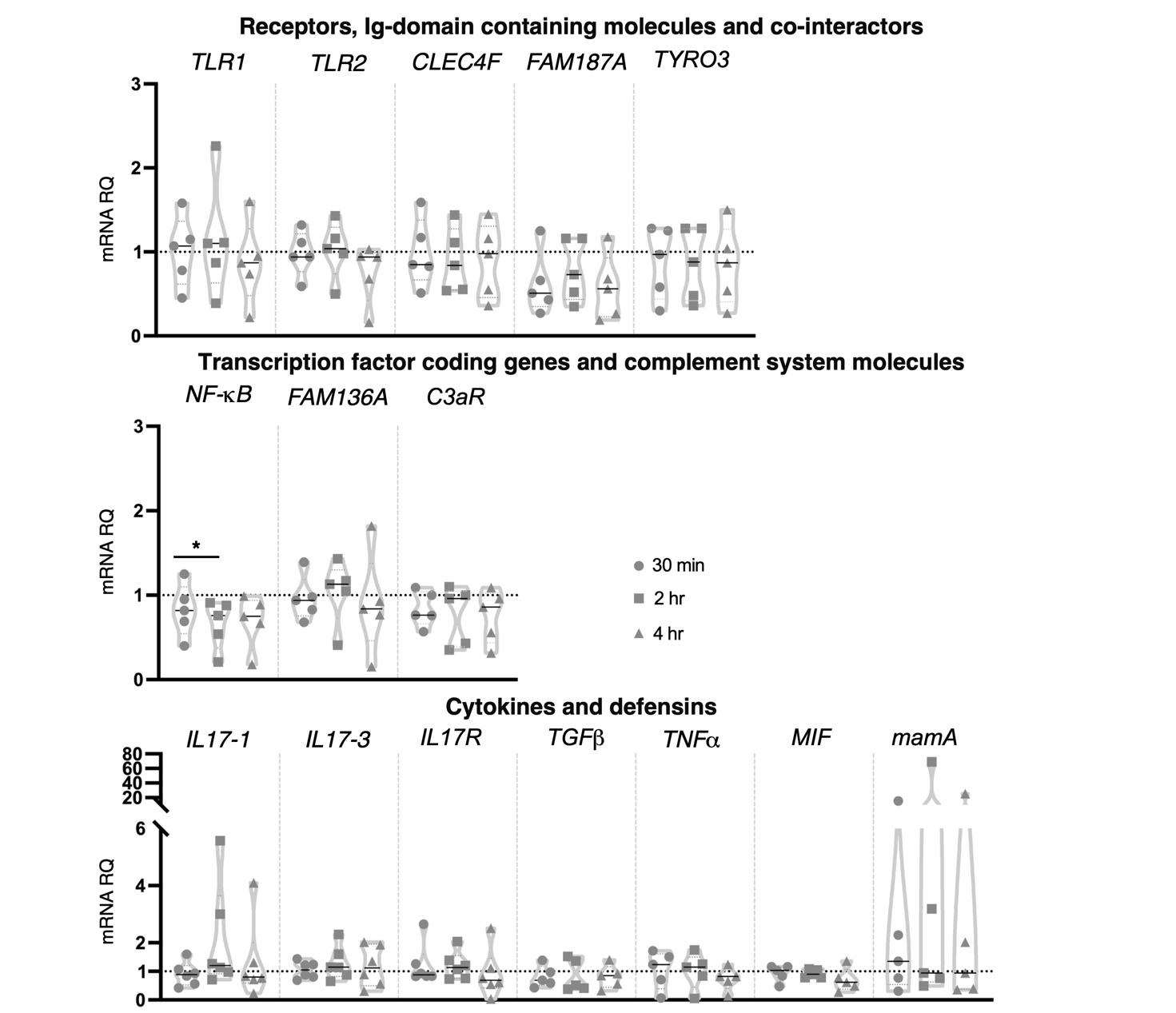


**Supplementary Figure 4: Genes not effected by treatment with 10 μg/ml Zymosan.** *C. robusta* juveniles treated with 10 μg/ml zymosan for 30 min, 2 hr and 4 hr, do not show significant changes in gene expression, measured by RT-qPCR, of *TLR1*, *TLR2*, *CLEC4F*, *FAM187A*, *TYRO3*, *NF-κB*, *FAM136A*, *C3aR*, *IL17-1*, *IL17-3*, *IL17R*, *TGFβ*, *TNFα*, *MIF*, and *mamA*. Truncated violin plots represent the distribution and the density of numerical data of gene expression reported as fold changes (2^-ΔΔCt^) of mRNA Relative Quantification (mRNA RQ) compared to the corresponding control samples of juveniles (not treated) and reported as dotted black line. The black lines in each violin plots indicate the median of data set (n = 5, biological replicates). Statistical methods: paired samples t-test and one-way ANOVA test.


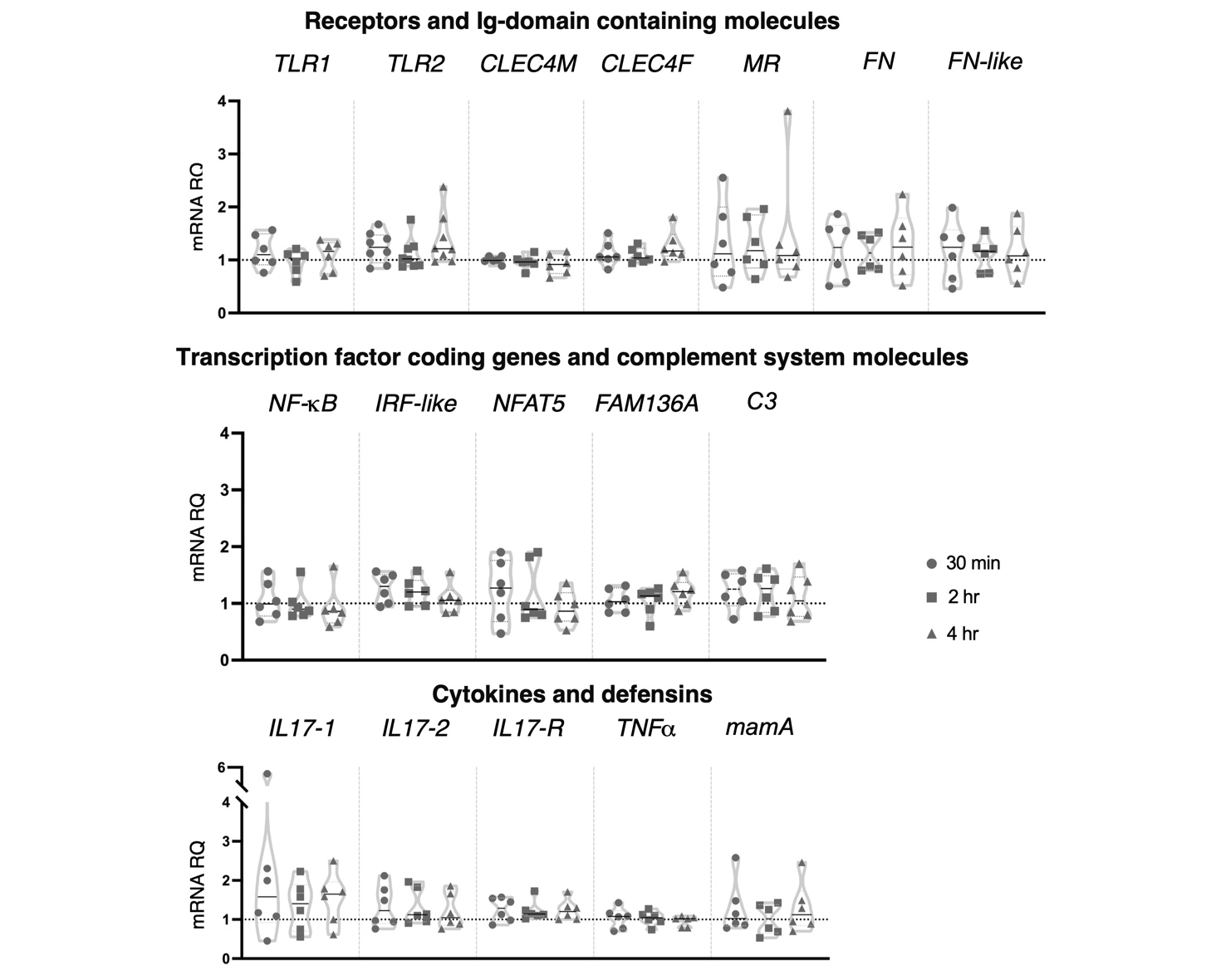


**Supplementary Figure 5: Genes not effected by treatment with 100 μg/ml Zymosan.** *C. robusta* juveniles treated with 100 μg/ml zymosan for 30 min, 2 hr and 4 hr, do not show significant changes in gene expression, measured by RT-qPCR, of *TLR1*, *TLR2*, *CLEC4M*, *CLEC4F*, *MR*, FN, *FN-like*, *NF-κB*, *IRF-like*, *NFAT5*, *FAM136A*, C3, *IL17-1*, *IL17-2*, *IL17-R*, *TNFα* and *mamA*. Truncated violin plots represent the distribution and the density of numerical data of gene expression reported as fold changes (2^-ΔΔCt^) of mRNA Relative Quantification (mRNA RQ) compared to the corresponding control samples of juveniles (not treated) and reported as dotted black line. The black lines in each violin plots indicate the median of data set (n = 6, biological replicates). Statistical methods: paired samples t-test and one-way ANOVA test.

**
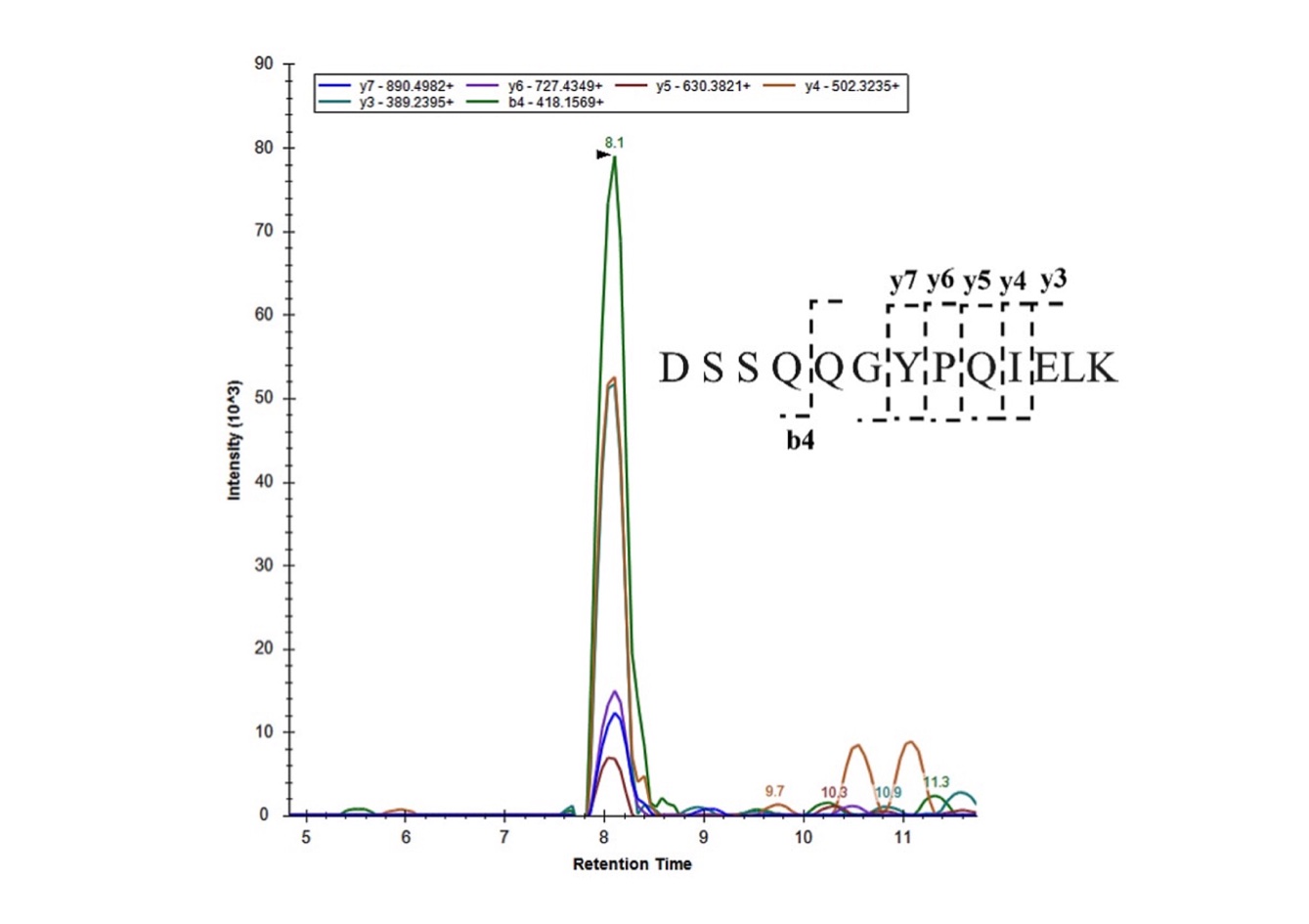
**

**Supplementary Figure 6: MRM chromatogram of the proteotypic peptide (DSSQQGYPQIELK)** **of nuclear factor of activated T-cells 5 (NFAT5) from *C. robusta.*** The reported transitions, such as 746.8 m/z → 890.5 m/z; 746.8 m/z → 727.4 m/z; 746.8 m/z → 630.4; 746.8 m/z → 502.3 m/z; 746.8 m/z → 389.2; 746.8 m/z → 418.1 m/z, were monitored for the selected peptide.

**
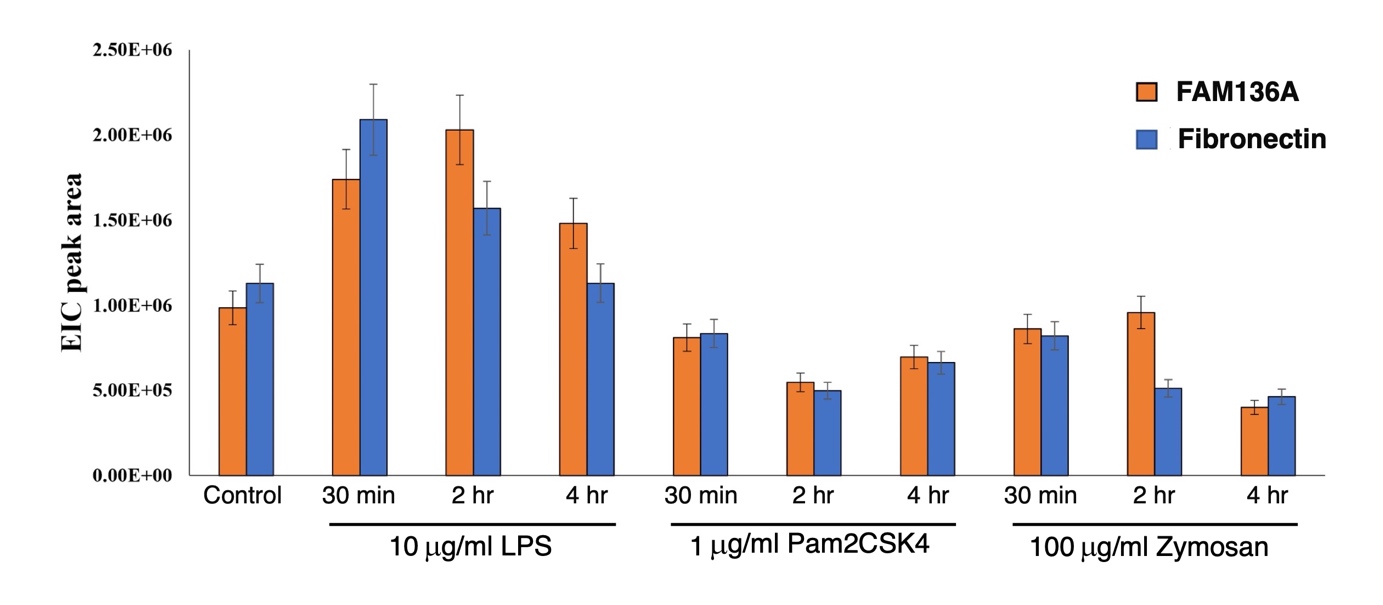
**

**Supplementary Figure 7:** **EIC peak areas recorded for the proteins FAM136A and Fibronectin**. Protein levels of FAM136A and FN are detected in different treatment conditions of *C. robusta* juveniles using the LC-MRM/MS method.

| Category | Gene Name | GenBank  Acc. Number | Oligonucleotide (5’-3’) | Amplicon (bp) | E |
| --- | --- | --- | --- | --- | --- |
| Receptors | **CLEC4M**  C-type lectin domain family 4 member M | XM_026833759.1 | F: gcaacagttggaatgcgacaaca  R: tcaccatcgtcccacaccca | 180 | 96% |
|  | **CLEC4F ***  C-type lectin domain family 4 member F | XM_002121194.4 | F: tcgctctgttcgaaaagcaaagat  R: acgcaaaggattttgaagaacggt | 120 | 96% |
|  | **MR ***  Macrophage mannose receptor 1-like. | XM_026835825.1 | F: ggtgatgaaagtactgctgcgtg  R: aacgagatccgcttgttgtgc | 114 | 100% |
|  | **TLR1**  Toll-like receptor 1 | NM_001166127 | F: tgtgaggagaaacgcttggt  R: ccatctcctacacaacgccaa | 128 | 100% |
|  | **TLR2**  Toll-like receptor 2 | NM_001166128 | F: tacgcaagtagtgcatggtgt  R: tctcttgtaacatcctccagca | 113 | 100% |
| Ig-domain containing molecules | **FAM187A**  Ig-like V-type domain-containing protein | XM_002120001.4 | F: atctccgctggcactactcg  R: cgccatgatgcaagcactct | 70 | 95% |
|  | **FN** *  Fibronectin. | KX766380 | F: tgctgtgatcgatggtacacg  R: cggtccctcggctgttattt | 151 | 100% |
|  | **FN-like**  Fibronectin-like | XM_002120240.4 | F: tggtcctcccgaaaatgttcaact  R: tgctgtgttcaaaggctcagatgt | 190 | 98% |
|  | **TYRO3 ***  Tyrosine-protein kinase receptor | XM_002124852.4 | F: tggggcgcgtttttgttctagt  R: tgtggggtaagacgggatagtgtt | 168 | 95% |
| Co-interactors | **SYK**  Tyrosine-protein kinase | XM_002122964.5 | F: cccacaacgcatcgagcaaa  R: tgtggccgttgagagacagg | 154 | 97% |
| Transcription factor coding genes | **IRF-like ***  Interferon regulatory factor-like | NM_001078275.1 | F: ccaatgtcctcgcttgccac  R: tgatgcgtatggctcgcagt | 167 | 97% |
|  | **NFAT5 ***  Nuclear factor of activated T-cells 5 | XM_018811319.2 | F: gcacgctatctcacagaggg  R: tcttcacaagcggtcgtgtt | 200 | 99% |
|  | **NF-κB ***  nuclear factor kappa b | NM_001078304 | F: tgtcgcttgtcgtcatggaa  R: aacacccaagaccgtcgaaa | 107 | 100% |
|  | FAM136A * | XM_002129844.5 | F: tgtgttttacaacgtagccggt  R: agtgtcgatgcgcttcagtg | 121 | 100% |

**Supplementary Table 1a: Primer sequence and efficiency of *Ciona robusta* immune genes.** List of *C. robusta* immune molecules, investigated by RT-qPCR experiments after microbial stimuli treatment, reporting gene accession number in NCBI, primers sequence and efficiency (E). Black asterisk indicates molecules that have been investigated through proteomic approach.

| Category | Gene Name | GenBank  Acc. Number | Oligonucleotide (5’-3’) | Amplicon (bp) | E |
| --- | --- | --- | --- | --- | --- |
| Cytokines | **IL17-1 ***  Interleukin 17-1 | NM_001129875.1 | F: aggttaagaatccctatggtgc  R: caaaggcacagacgcaaagg | 105 | 100% |
|  | **IL17-2** Interleukin 17-2 | NM_001129874 | F: ctcaaaacaggaaacaatgtcgaag  R: gcggtgctagtgcccatttt | 118 | 96 % |
|  | **IL17-3** Interleukin 17-3 | NM_001129876.1 | F: caccaagaccgtaataggcga  R: tttatgttttccgccagcgt | 101 | 100% |
|  | **IL17R ***  Interleukin 17 receptor | NM_001245045 | F: tgttggcatgagtgttcggt  R: agttggttctgccccaaagt | 103 | 96% |
|  | **MIF ***  Macrophage migration inhibitory factor | XM_002120701.4 | F: gtcgtttggtgggactgagga  R: gctgcgagttggtggaaagt | 213 | 96% |
|  | **TGFβ ***  Transforming growth factor-beta | NM_001078370.1 | F: ggggaaaggaaccaggttgaa  R: ccacacccgtgtccattagt | 106 | 100% |
|  | **TNFα ***  Tumor necrosis factor alpha | NM_001128107 | F ttcagaaagattggacgacga  R tcgtttagaaatgctgctgtgg | 106 | 100% |
| Complement system molecules | **C3**  Complement 3 | NM_001032512 | F: agcgaaagatccattggtac  R: gcaattcctggttccacagt | 174 | 100% |
|  | **C3aR ***  Complement 3  receptor | NM_001078552 | F: tggggactccacacaggaat  R: gtaccccctccgccg | 159 | 98% |
| Defensins | **mamA**  molecule against microbes A precursor | NM_001126229.1 | F: actccgcttaacgcatgcac  R: tggagcaaagttcaaagaagcagc | 85 | 98% |
| Reference gene | Cytoskeletal Actin | AJ297725 | F: ccggcttcagctgctaagta  R: tgggtattccgatgtattggtcc | 88 | 96% |

**Supplementary Table 1b: Primer sequence and efficiency of *Ciona robusta* immune genes.** List of *C. robusta* immune molecules, investigated by RT-qPCR experiments after microbial stimuli treatment, reporting gene accession number in NCBI, primer sequence and efficiency (E). Black asterisk indicates molecules that have been investigated through proteomic approach.

| **Sample** | **Component 1** | **Component 2** | **Component 3** | **Component 4** | **Component 5** |
| --- | --- | --- | --- | --- | --- |
| Control | -3.86 | 0.62 | 0.20 | -0.31 | -0.87 |
| 10 μg/ml LPS  30 min | -2.43 | -1.43 | -1.59 | 1.78 | 0.86 |
| 10 μg/ml LPS  2hr | -1.73 | -0.78 | -1.73 | -0.60 | -0.51 |
| 10 μg/ml LPS  4hr | -1.02 | -1.75 | -0.25 | -0.74 | -0.20 |
| 1 μg/ml Pam2CSK4 30 min | -0.94 | 0.19 | -0.50 | -1.33 | 0.70 |
| 1 μg/ml Pam2CSK4 2 hr | -1.49 | 3.04 | -0.01 | -0.28 | 1.08 |
| 1 μg/ml Pam2CSK4 4 hr | 14.65 | -0.11 | -0.14 | 0.06 | -0.06 |
| 100 μg/ml Zymosan 30 min | -1.34 | -0.98 | 0.45 | 0.49 | -0.96 |
| 100 μg/ml Zymosan 2 hr | -1.31 | -1.94 | 2.92 | 0.07 | 0.63 |
| 100 μg/ml Zymosan 4 hr | -0.54 | 3.15 | 0.65 | 0.86 | -0.67 |

**Supplementary Table 3: Principal component analysis (PCA).** PCA components (from 1 to 5) explaining the variation of expression of 16 proteins at each treatment condition.

| Protein acronym | Protein name | NCBI Protein  Accession Number |
| --- | --- | --- |
| AP complex | PREDICTED: AP-1 complex subunit sigma-2 | XP_002122331.1 |
| ANP32A | Acidic nuclear phosphoprotein 32 family member A | XP_002125600.1 |
| CLEC17A-like | PREDICTED: C-type lectin domain family 17, member A-like | XP_009859633.1 |
| COPS5 - COP9 | COP9 signalosome complex subunit 5-like | XP_002129245.1 |
| DHX36 | ATP-dependent RNA helicase DHX36 | XP_002120730.1 |
| DHX9 | ATP-dependent RNA helicase A | XP_009860016.1 |
| EF1-gamma-A-like | Elongation factor 1-gamma-A-like | XP_002131081.1 |
| FUT13 | Fut13 protein precursor | NP_001027702.1 |
| GOT1 | PREDICTED: Aspartate aminotransferase, cytoplasmic-like | XP_002129160.1 |
| GOT2 | Aspartate aminotransferase, mitochondrial | XP_002126101.1 |
| HPDL_X1 | PREDICTED: 4-hydroxyphenylpyruvate dioxygenase isoform X1 | XP_009862491.1 |
| HPDL-like | PREDICTED: 4-hydroxyphenylpyruvate dioxygenase-like partial | XP_002125704.1 |
| Hsp70 | Heat shock protein 70 | NP_001029006.1 |
| IKK-β | Inhibitor of nuclear factor kappa-B kinase subunit beta isoform X1 | XP_002125567.1 |
| IRAK-4-like | Interleukin-1 receptor-associated kinase 4-like | XP_002128882.1 |
| IRAK4 | PREDICTED: Interleukin-1 receptor-associated kinase 4 isoformX1 | XP_002122012.1 |
| ITGA8 | PREDICTED: Integrin alpha-8 | XP_002123970.1 |
| IκB | IkappaB protein | NP_001071739.1 |
| MAPKK3/6 | Mitogen-activated protein kinase kinase 3/6 | NP_001071941.1 |
| MAPKK4 | Mitogen-activated protein kinase kinase 4 | NP_001071758.1 |
| MBL-like | C-type lectin mannose-binding isoform-like | XP_002129025.1 |
| Monocyte to macrophage differentiation factor | Monocyte to macrophage differentiation factor | XP_002127427.1 |
| MR-1-like | PREDICTED: macrophage mannose receptor 1-like, partial | XP_002124779.1 |
| MR1-like (LOC100186027) | uncharacterized protein LOC100186027, possible (predicted) MR1-like | XP_002123456.2 |
| MRPS29 | 28S ribosomal protein S29, mitochondrial-like | XP_009859583.1 |
| MRPS5 | PREDICTED: 28S ribosomal protein S5, mitochondrial-like | XP_002121440.2 |
| MyD88 | PREDICTED: myeloid differentiation primary response protein MyD88 | XP_002121702.3 |
| p38 | Mitogen-activated protein kinase; P38 kinase | NP_001071958.1 |
| PAPILIN-like | Papilin-like | XP_002121111.1 |
| REL1 | PREDICTED: rel1 protein isoform X1 | XP_009860199.1 |
| RPL18a-like | 60S ribosomal protein L18a-like | XP_002122365.1 |
| RPL23 | 60S ribosomal protein L23 | XP_002121204.3 |
| RRP46_X1 | PREDICTED: exosome complex component RRP46 isoform X1 | XP_002130542.1 |
| SSPO | PREDICTED: SCO-spondin | XP_009861234.1 |
| TAK1 | Mitogen-activated protein kinase kinase kinase | NP_001071829.1 |
| TAT | Tyrosine aminotransferase | XP_002120196.1 |
| TGFβ/MAP3K7.1-like | PREDICTED: TGF-beta-activated kinase 1 and MAP3K7-binding protein 1-like | XP_002122073.1 |
| TGFβ/MAP3K7.2-like | PREDICTED: TGF-beta-activated kinase 1 and MAP3K7-binding protein 2-like | XP_002128681.2 |
| TMCO4-like | Transmembrane and coiled-coil domain-containing protein 4-like | XP_002126686.1 |
| TNR-like | PREDICTED: tenascin-R-like | XP_002124787.2 |
| Tollipb | PREDICTED: Toll-interacting protein B | XP_002126681.1 |
| TRAF3 | TNF receptor-associated factor 3 | XP_002121961.1 |
| TRAF3-like | PREDICTED: TNF receptor-associated factor 3-like | XP_009862001.1 |
| USP15 | Ubiquitin carboxyl-terminal hydrolase 15 | XP_002128421.1 |
| ZNF | Zinc finger protein | NP_001071979.1 |

**Supplementary Table 4: *Ciona robusta* proteins included in the interaction network.** Acronym, full name, and NCBI protein accession number of proteins present in the protein-protein interaction network but not investigated by transcriptional or proteomic analysis.
